# New palladium–oxazoline complexes: Synthesis and evaluation of the optical properties and the catalytic power during the oxidation of textile dyes

**DOI:** 10.3762/bjoc.11.132

**Published:** 2015-07-15

**Authors:** Rym Hassani, Mahjoub Jabli, Yakdhane Kacem, Jérôme Marrot, Damien Prim, Béchir Ben Hassine

**Affiliations:** 1 Laboratoire de Synthèse Organique, Asymétrique et Catalyse Homogène (11URES56), Faculté des sciences de Monastir, Avenue de l’Environnement, 5019 Monastir, Tunisia, Tel: 0021673500279, Fax: 0021673500278; 2 University of Versailles Saint-Quentin-en-Yvelines, Institut Lavoisier de Versailles, UMR CNRS 8180, 45, avenue des Etats-Unis, 78035 Versailles, France

**Keywords:** aminoalcohols, catalysis, dye decolorization, optical properties, oxazolines, palladium complexes

## Abstract

The present paper describes the synthesis of new palladium–oxazoline complexes in one step with good to high yields (68–95%). The oxazolines were prepared from enantiomerically pure α-aminoalcohols. The structures of the synthesized palladium complexes were confirmed by NMR, FTIR, TOFMS, UV–visible spectroscopic analysis and X–ray diffraction. The optical properties of the complexes were evaluated by the determination of the gap energy values (*E*_g_) ranging between 2.34 and 3.21 eV. Their catalytic activities were tested for the degradation of Eriochrome Blue Black B (a model of azo dyes) in the presence of an ecological oxidant (H_2_O_2_). The efficiency of the decolorization has been confirmed via UV–visible spectroscopic analysis and the factors affecting the degradation phenomenon have been studied. The removal of the Eriochrome reached high yields. We have found that the complex **9** promoted 84% of color elimination within 5 min (*C*_0_ = 30 mg/L, *T* = 22 °C, pH 7, H_2_O_2_ = 0.5 mL) and the energetic parameters have been also determined.

## Introduction

Palladium complexes have been used as starting materials to prepare polymers [1], agrochemicals [2], pharmaceuticals [3], flavors and fragrances [4]. They have also been used for the total synthesis of natural products and nanocompounds [5]. It is only since 1986 that oxazoline-based ligands have been utilized in asymmetric catalysis. This initiated considerable research activity in this field and triggered the synthesis of numerous chiral ligands containing at least one oxazoline ring [6]. Oxazoline units are expected to readily coordinate to a metal center and have been shown to bind a wide range of transition metals [7]. The wide variety of ligands with one or more oxazoline rings incorporating different heteroatoms, additional chiral groups, and specific structural features have been used in diverse reactions [8]. The importance of these ligands lies in the fact that the stereogenic centers are kept in close proximity to the metal and thereby having a strong and direct influence on the stereochemical course of the metal-catalyzed process.

Oxazolines possess several interesting advantages: versatility of the ligand design, their straightforward synthesis from readily available starting materials and variability of the chiral center, which are located near the donor atoms. The oxazoline unit is anticipated to increase the stability of a metal complex giving rise to more air- and possibly water-stable catalysts [9].

Commonly, difficulties arise when trying to treat waste waters containing dyes because the dyes are recalcitrant molecules, often resistant to aerobic digestion, and stable to light, heat, and oxidizing agents [10–11]. Recently, the catalytic oxidation was recognized as an effective method to treat colored waters [12].

In fact, the treatment of colored waters remains a serious environmental topic. Many industries such as textile, leather and paper discharge various dyes during their processing operations [13–14]. These dyes are toxic, mutagenic, and carcinogenic [15–16]. That’s why many unconventional methods and techniques have been investigated and a number of studies have been developed [17–19]. In particular, attention has been focused on the synthesis of supports having metal complexes in their structures due to their capacities and efficiencies to treat colored waters [20–22].

Owing to the easy formation of palladium–oxazoline complexes, it was very interesting to investigate the ability of some synthetic dyes to coordinate to palladium complexes by developing binary systems. This was done by complexing oxazoline with Pd^II^ ions, leading to the adsorption of dyes. Moreover, the decolorization of the solution by Pd and Pd complexes has also been applied [23].

**Figure 1:**
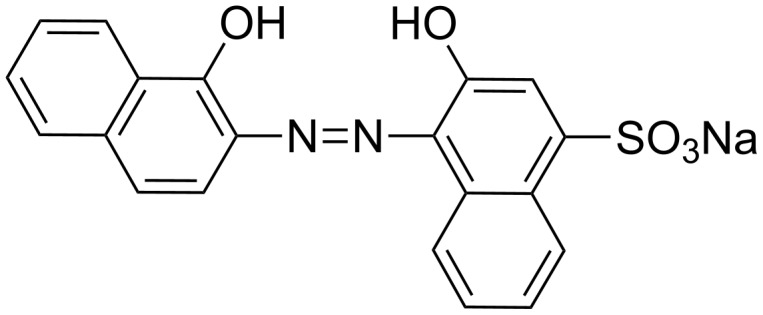
Structure of Eriochrome Blue Black B.

In this paper, we report the synthesis and characterization of some new palladacycles. Optical properties were determined and the catalytic activity of these complexes was evaluated for the first time during the degradation of organic dyes such as Eriochrome Blue Black B referred to as Erio which was chosen as an example of azo dyes (Figure 1).

## Results and Discussion

The use of oxazolines as ligands during the preparation of optically active cyclopalladated compounds can give rise to different applications [24–28].

The compilation of the literature shows that the presence of substituents on the ligand promotes the cyclopalladation. Smoliakova and co-workers performed the cyclopalladation of *tert*-butyl-substituted phenyloxazoline by stirring the reaction mixture at room temperature [29].

**Scheme 1:**
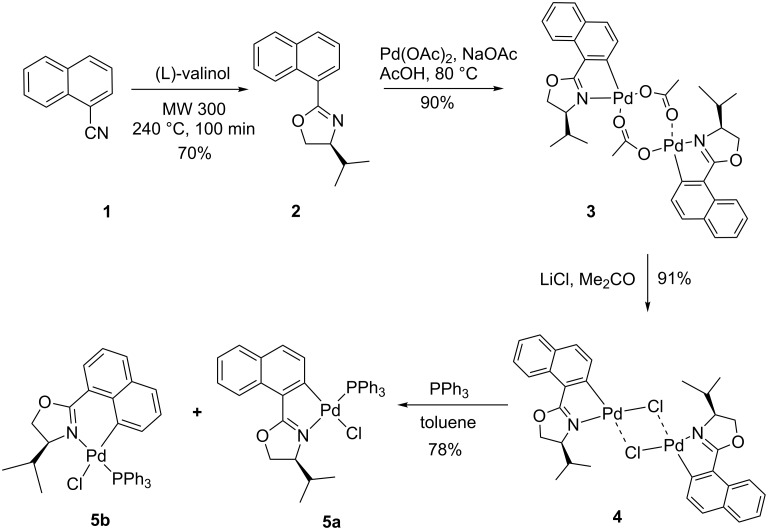
Cyclopalladation reactions of (*S*)-4-isopropyl-2-(naphthalen-1-yl)oxazoline.

In the light of these results, we also attempted to carry out this process under milder conditions. When (*S*)-4-isopropyl-2-(naphthalen-1-yl)oxazoline (**2**) was added to an acetic acid solution of Pd(OAc)_2_, a yellowish precipitate was obtained and identified as (*S*,*S*)-di-μ-acetatobis[1-(4-isopropyloxazolin-2-yl)naphthalen-2-yl-*C*,*N*]dipalladium(II) (**3**) (Scheme 1). Unfortunately, complex dimer **3** was relatively unstable, so only its ^1^H NMR and FTIR data were performed. The metathesis of dimer **3** with lithium chloride in acetone afforded the more stable (*S*,*S*)-dimer **4** in which NMR analysis shows the presence of two dimeric forms. For better elucidation of their structures, dimeric complexes **4** were transformed into their mononuclear phosphane derivatives **5a** and **5b** using PPh_3_ in toluene. According to ^1^HNMR data, the mixture contains **5a** and **5b** in the ratio of 4:1. Essays to separate the later regioisomers by column chromatography or preparative TLC on silica gel were unsuccessful.

According to the literature, the PPh_3_ adducts of cyclopalladated oxazolines have the *trans* (*N,P*) geometry [30–31]. In addition, the palladacycles with oxazoline ligands exists in *endo* and *exo* regioisomers. Results obtained in the present study show that naphthyl-oxazoline has a tendency to form exclusively *endo*-cyclic complexes with the C=N bond within a palladacycle. Furthermore, NMR data analyses demonstrate that the five-membered palladacycle **5a** was preferentially formed upon the six-membered **5b**. Indeed, palladium insertion in the *peri* C–H bond of the naphthyl-oxazoline will take place at high temperature reactions [32].

In order to investigate the effect of solvents on the isomeric ratio, cyclopalladation of oxazoline **2** was also carried out with Pd(OAc)_2_ in refluxing MeCN. After 3.5 h the reaction yielded 89% of the expected dimer. However, a significant change on the isomer ratio was observed.

**Scheme 2:**
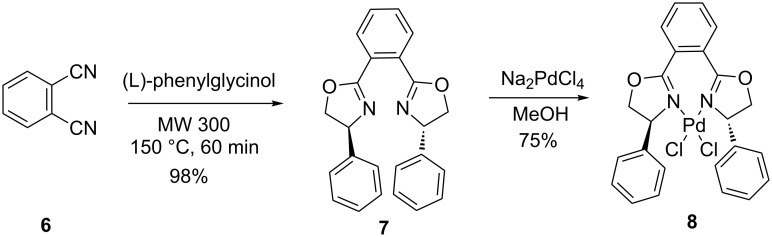
Synthesis of cyclopalladated complex from bis-oxazoline.

**Figure 2:**
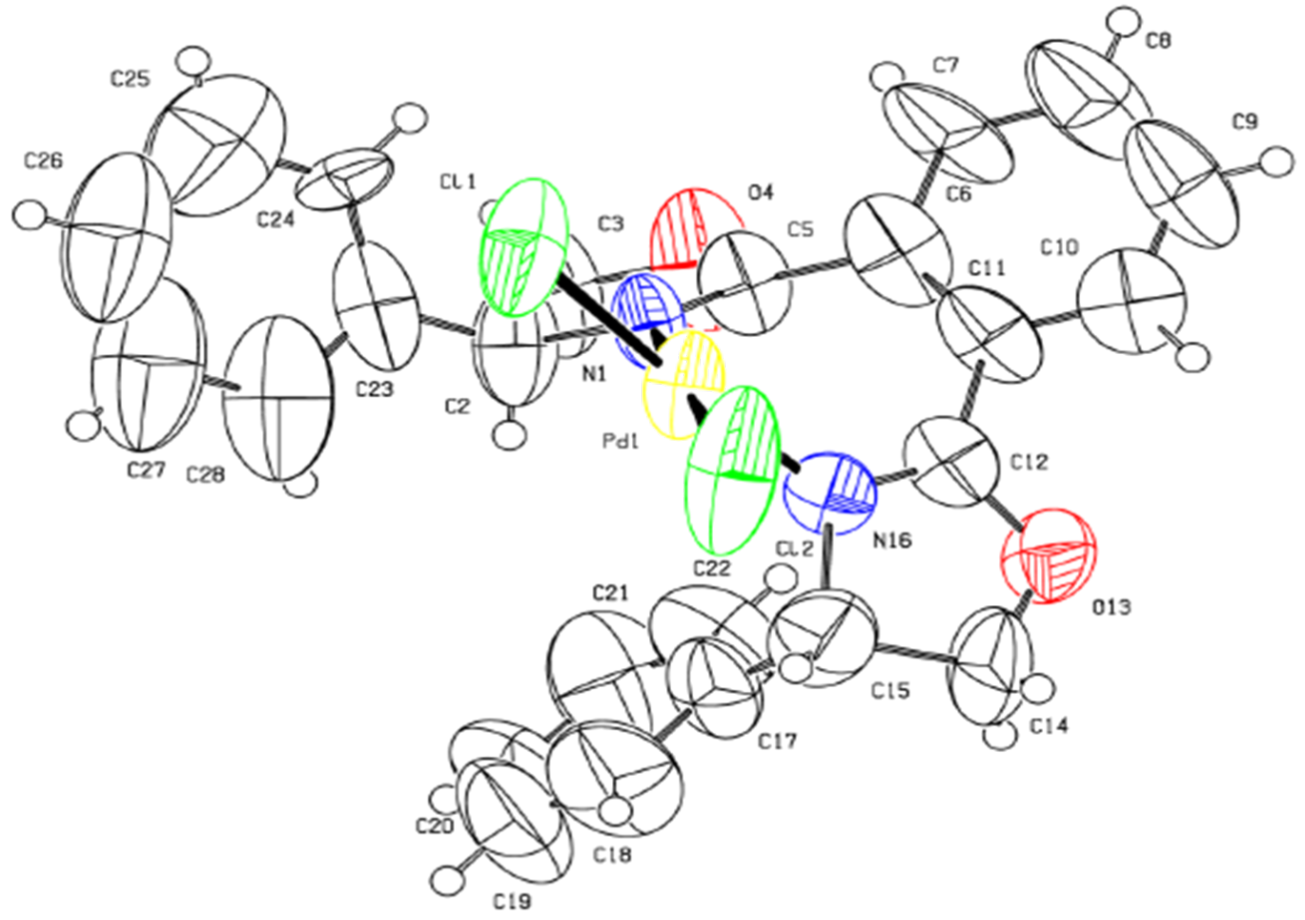
ORTEP drawing of the complex **8**.

In this work, we report one example related to the direct palladation of enantiopure bis-oxazoline **7** having a chiral center at the 4-position and the substituent at the 2-position of the heterocycles. The addition of one equivalent of **7** to a methanolic solution of Na_2_PdCl_4_ gave the palladium complex **8** in 75% yield. The coordination sphere has two chlorine atoms bonding in a *cis* configuration to the Pd center and the oxazoline ligand chelating to the Pd center via the two nitrogen atoms to form a planar palladium ring system (Scheme 2). Crystallization by slow diffusion of pentane into a solution of **8** in dichloromethane gave suitable crystals for an X-ray diffraction study. The molecular structure is presented in Figure 2 (see Supporting Information File 1 for the cif of complex **8**). The coordination geometry of **8** is pseudo-square planar with the four coordination sites occupied by the two nitrogen donors and the two chlorine atoms. The molecule possesses approximate *C*_2_ symmetry and its chirality has been confirmed to be the (*S*,*S*)-isomer. The Pd–N and the Pd–Cl distances (Pd–N16 distance (1.963 A°), Pd–N1 distance (2.031 A°)) are almost the same (Pd–Cl1 distance (2.281 A°), the Pd–Cl2 distance (2.259 A°)).

**Scheme 3:**
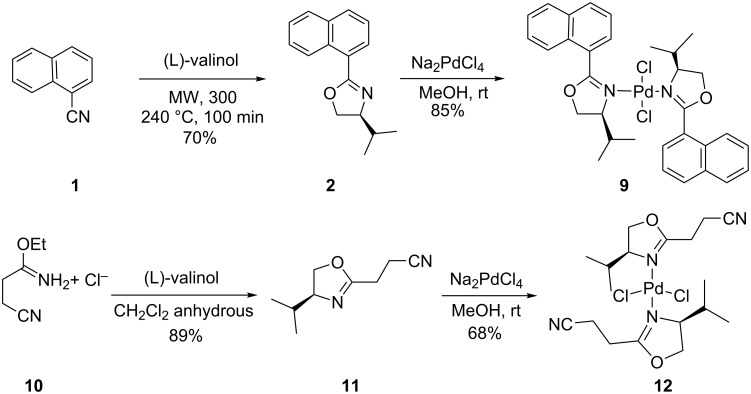
Synthesis of the bis(oxazoline) coordinated complexes.

**Figure 3:**
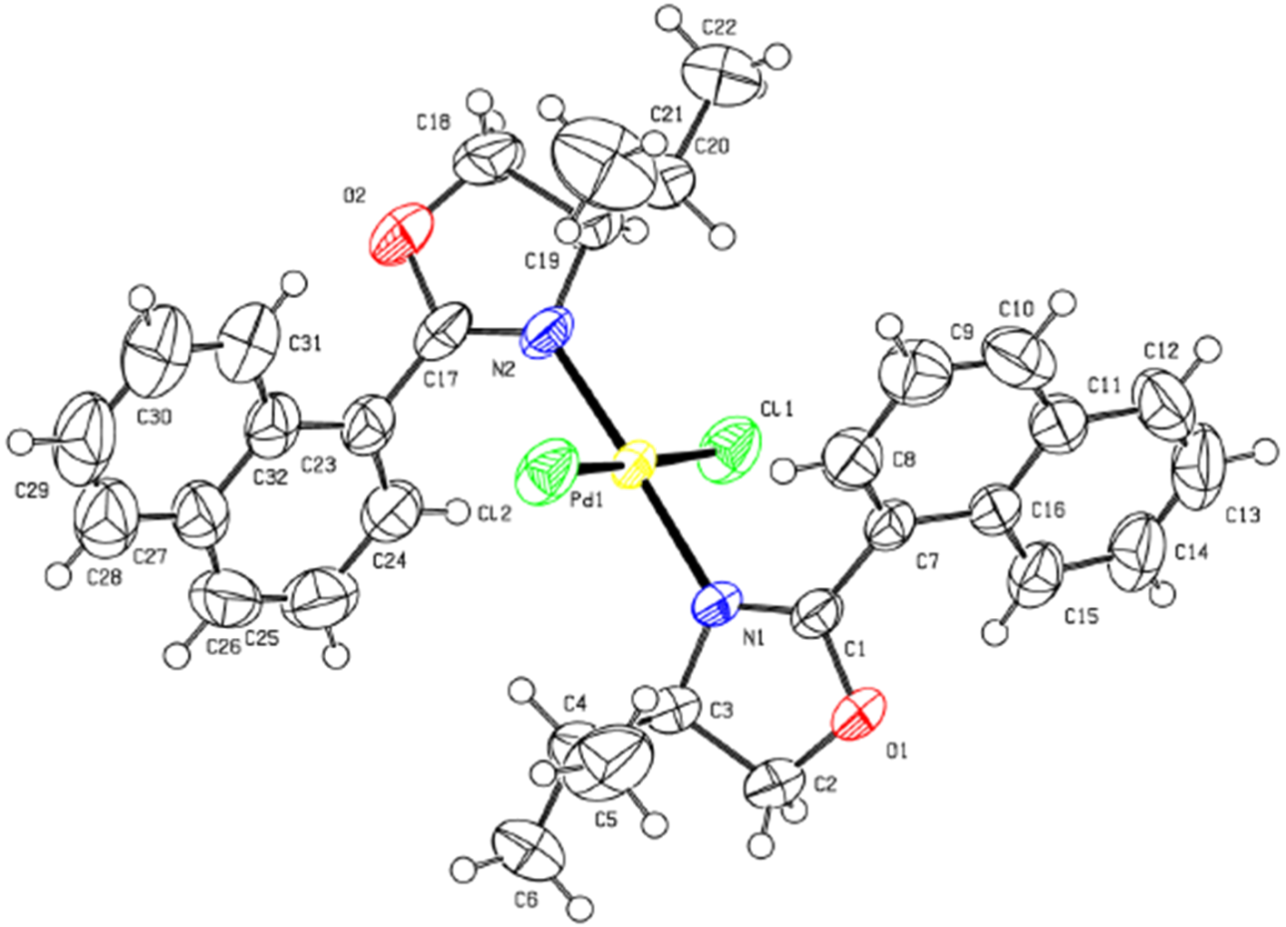
ORTEP drawing of the complex **9**.

On the other hand, the addition of (*S*)-4-isopropyl-2-(naphthalen-1-yl)oxazoline (**2**) and 3-[(4*S*)-4,5-dihydro-4-isopropyl-1,3-oxazol-2-yl]propanenitrile (**11**) to a methanolic solution of Na_2_PdCl_4_ at room temperature gave the palladium complexes **9** and **12** in 85% and 68% yields, respectively (Scheme 3). The two complexes are stable when exposed to air and water. The single crystal X-ray model of complex **9** is depicted in Figure 3 (see Supporting Information File 2 for the cif of complex **9**). This structure confirms the expected monomeric nature of the complex and coordination of the oxazoline nitrogen to the palladium atom. The Pd(II) unit is coordinated to two monodentate ligands with the two nitrogen and two chlorine atoms in equatorial positions to complete the distorted tetragonal coordination sphere. The two ligands coordinate to the palladium center in a *trans* geometry with respect to each other.

All the complexes prepared in this work were characterized by IR, ^1^H, ^13^C, 2D NMR, X-ray diffraction (for **9** and **8**) and UV–visible spectroscopy. The characterized bands in the IR spectra of all complexes are given in Table 1.

**Table 1:** The FTIR analysis of the complexes.

			vibration	bands ν (cm^−1^)		
attribution	**3**	**4**	**5**	**8**	**9**	**12**

ν(C–H)	2956.4	2954.8	2956.7	2961.7	2957.8	2963.9
ν(nitrile)	–	–	–	–	–	2250.8
ν(C=N)	1645.4	1641.6	1637.4	1638.2	1642.2	1635.4
ν(C–N)	1350.2	1355.3	1436.7	1345.7	1377.8	1545.2
δ(CH_3_)	1205.2	1210.0	1201.0	–	1200.4	1260.3
ν(P–C)	–	–	1094.9	–	–	–
ν(C–O)	1016.1	1012.9	1011.0	1023.2	1030.8	1018.8
δ(C–H)	760	766.2	692.3	773.6	776.0	–
δ(Pd–N)	–	510.9	513.4	525.2	573	–

### Optical properties

The gap energy values (*E*_g_) of the complexes determined from the absorption edge of the solution spectra are given in Table 2. The gap energy was evaluated by the extrapolation of the tangent to the first inflexion point of the UV curve.

**Table 2:** Optical properties of the chiral complexes.

complexes	λ_max_ (nm)	*E*_g_ (eV)

3	342	2.67
4	341	2.50
5	299	2.34
8	380	2.72
9	341	2.57
12	300	3.21

Finally, we have studied the catalytic activities of the new complexes during the decolorization of azo dyes in solutions which are discharged in textile industry.

### Oxidative degradation of dyes

**Figure 4:**
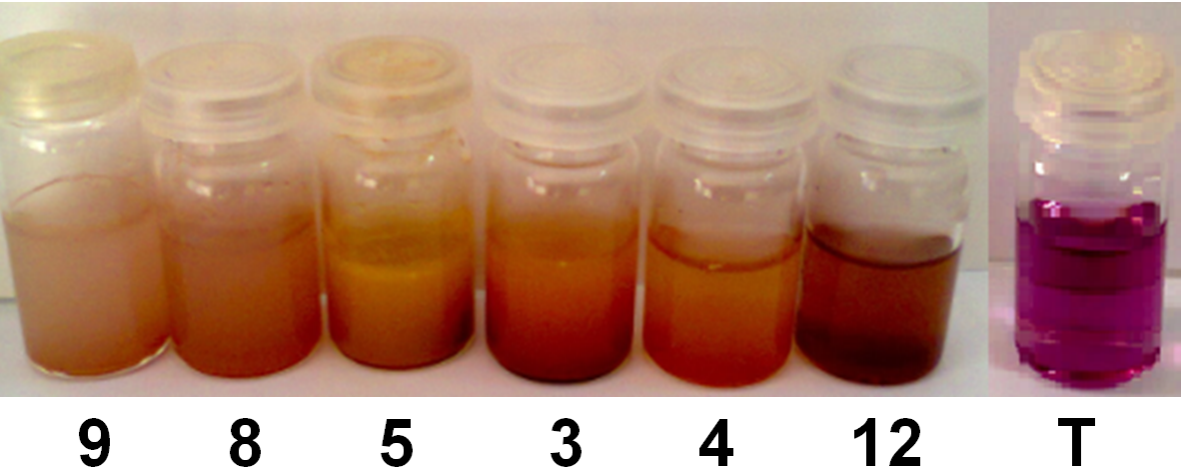
Change in color removal in the presence of different catalysts within 10 min (before filtration). T: sample which contain only the solution of dye and H_2_O_2_.

**Figure 5:**
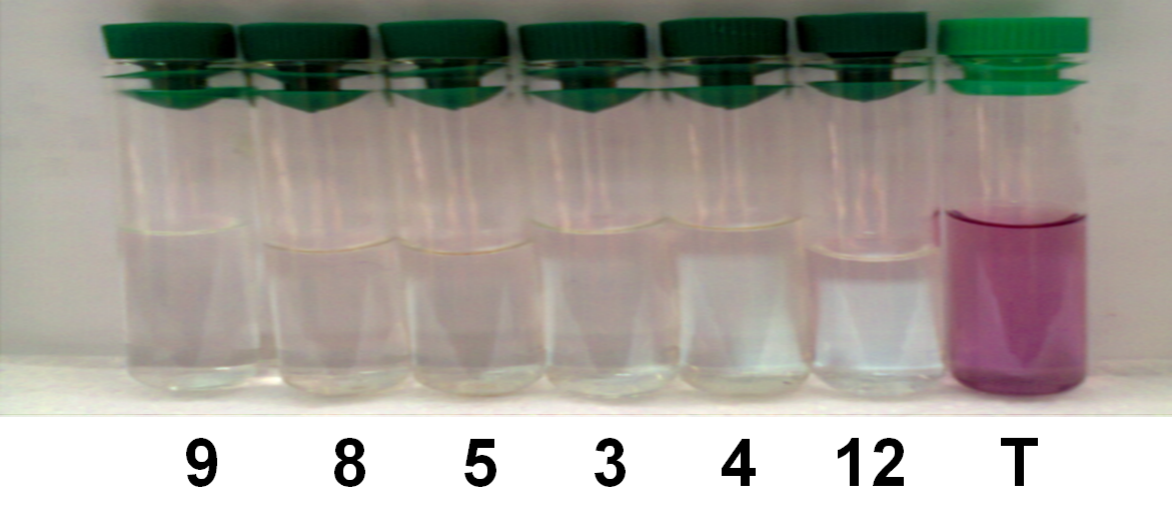
Change in color removal in the presence of different catalysts (after filtration over 10 min).

Six complexes were checked for the oxidative degradation of Eriochrome Blue Black B. The experimental results indicated that the complexes have potential activities during the degradation of the azo dyes in the aqueous medium and in the presence of hydrogen peroxide. From the preliminary data, it was found that all the prepared complexes have demonstrated a promising catalytic activity at the same conditions (*t* = 10 min, *C*_0_ = 30 mg/L, 10 mg of the catalyst, amount of H_2_O_2_ = 0.5 mL). Among the six compounds, catalyst **9** was found to be the most active during this study because the corresponding solution became almost colorless within five minutes (Figure 4). As also clearly depicted in Figure 5, the complete removal of Eriochrome was reached in 10 min for all samples tested.

In attempts to check the efficiency of the prepared complex **9** concerning the degradation of Eriochrome Blue Black B, we have discussed the results by varying the experimental conditions (contact time, initial dye concentration, temperature and H_2_O_2_ dose).

**Figure 6:**
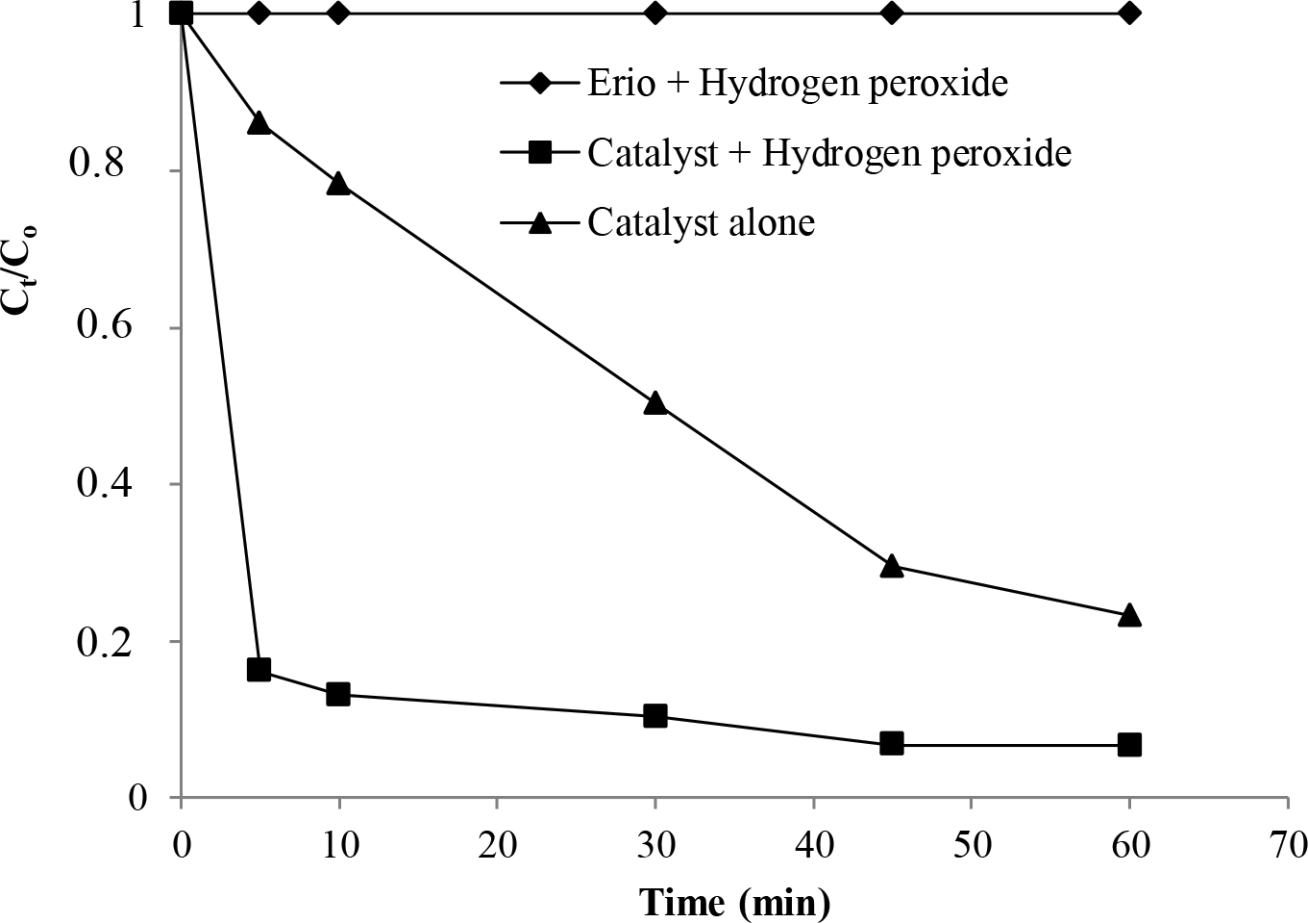
Evolution of the color degradation against time using Eriochrome plus H_2_O_2_, the complex plus H_2_O_2_ or the complex alone.

Figure 6 represents the evolution of the rate of the degradation of Eriochrome against time using H_2_O_2_, the complex **9** plus H_2_O_2_ and the complex alone [18]. First, it can be registered that the color solution remains persistent and stable in the presence of H_2_O_2_ without the addition of any catalyst. However, it was observed that the concentration of the dye declined sharply, in the presence of the system catalyst/H_2_O_2_. Indeed, 84% of the target was achieved in the presence of the prepared catalyst after only 5 min of reaction time at 22 °C whereas the dye removal does not exceed 14% using the complex alone. The efficiency of the combination of catalysts/H_2_O_2_ for the degradation of the studied azo dye is so confirmed and catalyst **9** is able to decompose the reaction products completely by the cleavage of the azo linkage (chromophore structure: –N=N–, responsible for the color) [33–34].

### Effect of the hydrogen peroxide concentration

As proved in the previous section, the action of H_2_O_2_ alone did not show any degradation capacity for the studied dye solution, although this agent is considered a relatively powerful oxidant. In this section, we examine the effect of H_2_O_2_ dose on the rate of dye removal for an initial dye concentration of 30 mg/L using 10 mg of the catalyst. Data given by Figure 6 revealed that the presence of the catalyst, with the increment in H_2_O_2_ concentration, significantly reduced the time necessary to decolorize the solution with high target removal. As an example, we observe that after only a 5 min reaction and at the optimum concentration of H_2_O_2_ (0.5 mL), the decolorization of the Eriochrome solution reached 84% whereas the target removal is about 18% for the studied catalyst in the same condition and using 0.2 mL of H_2_O_2_. This means that the increase of the oxidant concentration generates more free-hydroxyl radicals, causing the dye decolorization. On the contrary, a slight decrease of the decolorization process from 84% to 79% has occurred for a highest amount of H_2_O_2_ (0.7 mL) because the solution undergoes self-quenching (scavenger) of OH^·^ radicals by adding amounts of H_2_O_2_ to produce HO_2_^·^ radicals, according to the following equations:

H_2_O_2_ + HO^·^ → H_2_O + HO_2_^·^

HO_2_^·^+ HO^·^ → H_2_O_2_ + O_2_

**Figure 7:**
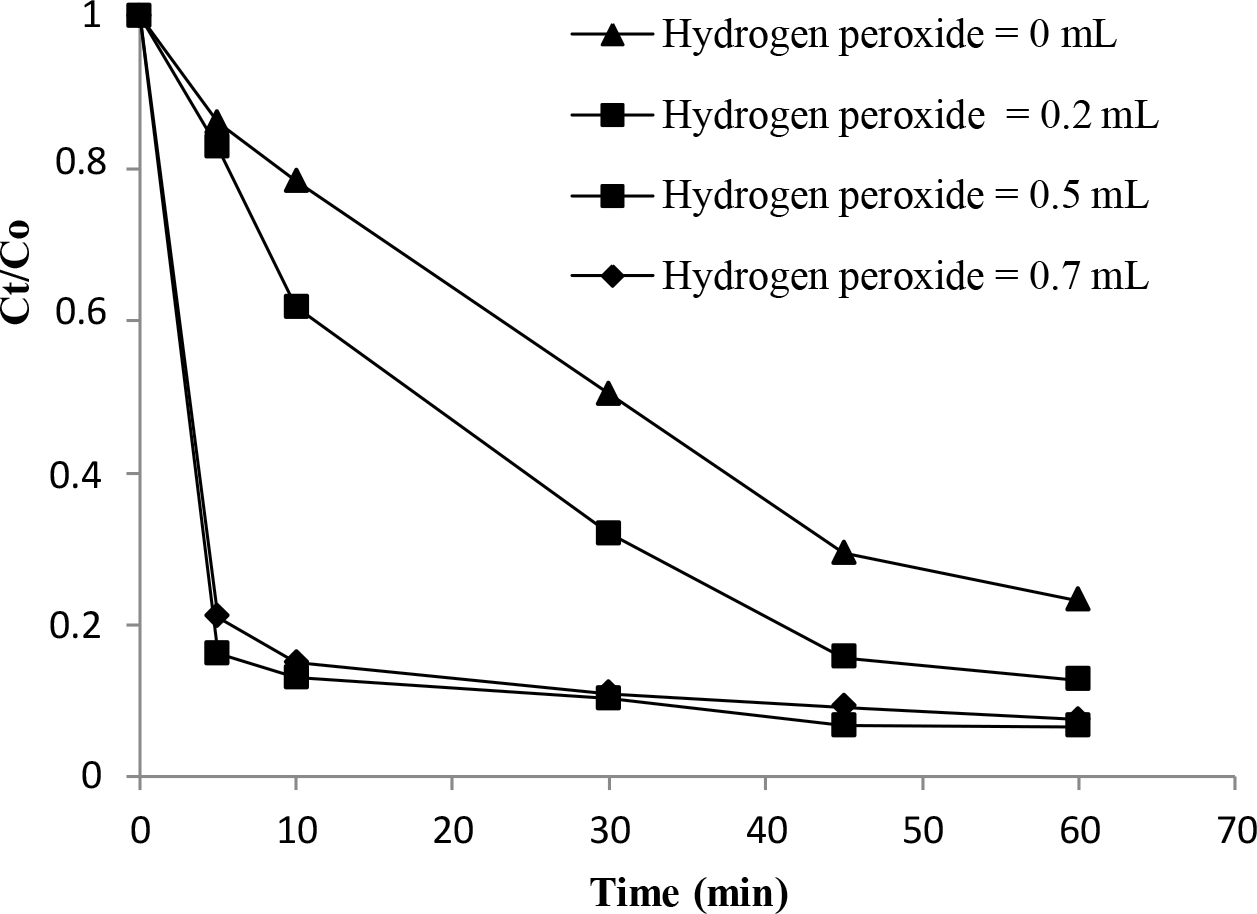
Change of the concentration of the Erio solution with the variation of H_2_O_2_ dose.

We observe that all curves depicted in Figure 7 have a pseudo-first-order kinetics shape, suggesting, therefore, a first-order kinetics law.

### Effect of the initial dye concentration on the decolorization process

**Figure 8:**
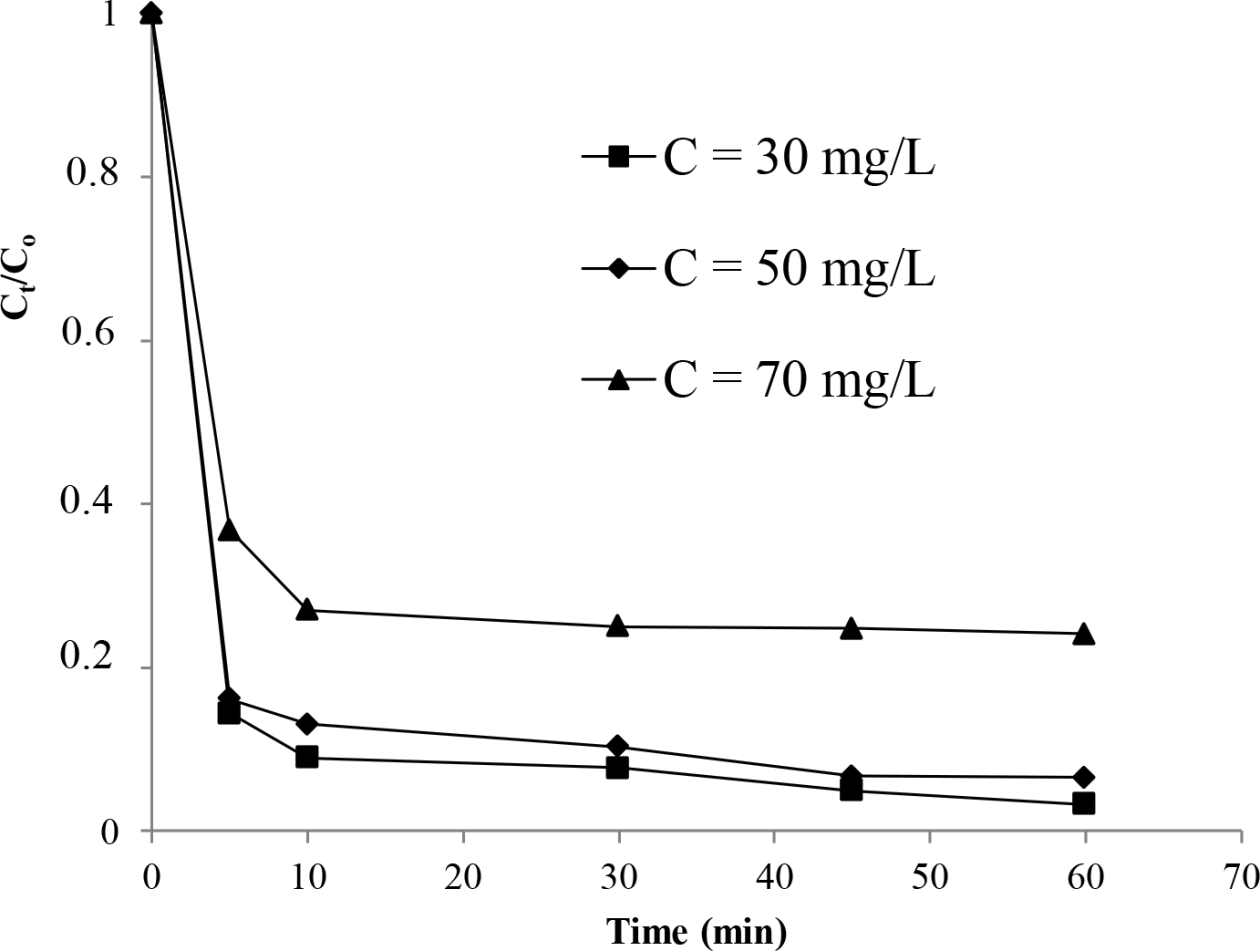
Evolution of the color removal against initial dye concentration.

The effect of the initial dye concentration on the decolorization process was studied at a constant dose of H_2_O_2_ and at a temperature of 22 °C. Data given in Figure 8 exhibited that the percentage of color removal decreased with the increase of the initial dye concentration. As an example, it decreased from 84% (C = 30 mg/L) to 64% (C = 70 mg/L) for a reaction time of 5 min in the presence of the studied catalyst. This confirms the fact that the decolorization process depends, on the concentration of H_2_O_2_ and the initial dye concentration. These trends were in agreement with those observed in our laboratory during the study of the degradation of azo dyes [18–20] and also with literature [35–36].

### Effect of the temperature on the decolorization process of dyes

**Figure 9:**
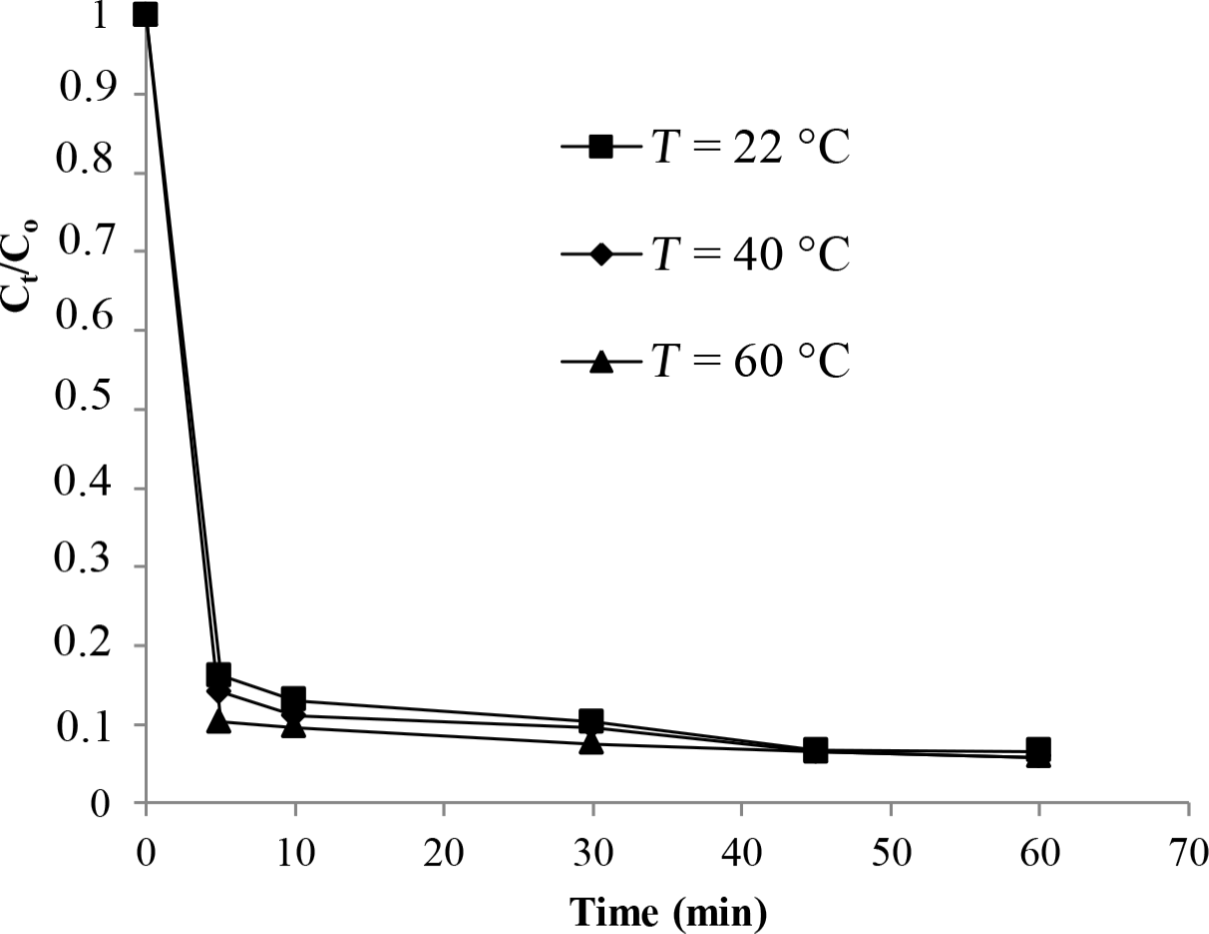
Change of the color removal versus temperature.

In this section, the effect of the temperature was studied at a constant dose of H_2_O_2_ and an initial dye concentration of 30 mg/L. The results shown in Figure 9 indicated that the removal of the color was enhanced when the temperature increased. The target removal after 5 min of reaction at 22 °C and 60 °C were, respectively, 84% and 94%, in the presence of the studied catalyst.

### Thermodynamic parameters

To better understand the degradation process, the pseudo first-order kinetic equation was used to determine the kinetic parameters. The Arrhenius law was used to calculate the activation energy (*E*_a_*)*. The determination of the entropy and the enthalpy of activations (ΔS* and ΔH*) were performed using the Eyring equation [37]. The results are summarized in Table 3.

**Table 3:** Calculation of the activation parameters for the dynamic process.

*T* (°C)	*k*_0_ (min^−1^)	*E*_a_ (kJ mol^−1^)	ΔS* (J mol^−1^ K^−1^)	ΔH* (kJ mol^−1^)	ΔG*(kJ mol^−1^)

22	0.0167				82.385
40	0.0211	16.669	−231.594	14.065	86.553
60	0.0363				91.185

The activation energy (*E*_a_) has been found to be low (16.669 kJ mol^−1^) confirming that the complex **9** was very efficient for the degradation of azo dyes using H_2_O_2_.

### Reuse of the catalysts

**Figure 10:**
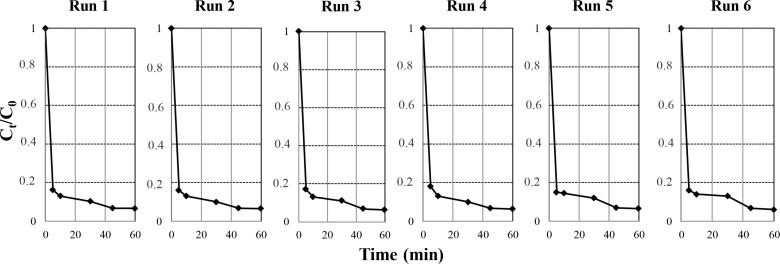
Recycling experiments for Erio removal (*C*_0_ = 30 ppm, 20 mL) in the presence of catalyst **9** at pH 7 and *T* = 22 °C.

In this section, the reusability of the catalysts was considered. To do this, for example, the catalyst **9** was separated by filtration, washed with distilled water after each run, then dried and further subjected to subsequent runs under the same conditions. The data given in Figure 10 indicate that the regeneration process could be repeated for six cycles, without appreciable activity loss.

The reuse of the prepared catalysts is found to be possible after separation and washing. The regenerated catalysts were also characterized by FTIR analyses after each cycle, and no change was observed.

### Suggested mechanism of decolorization

**Scheme 4:**
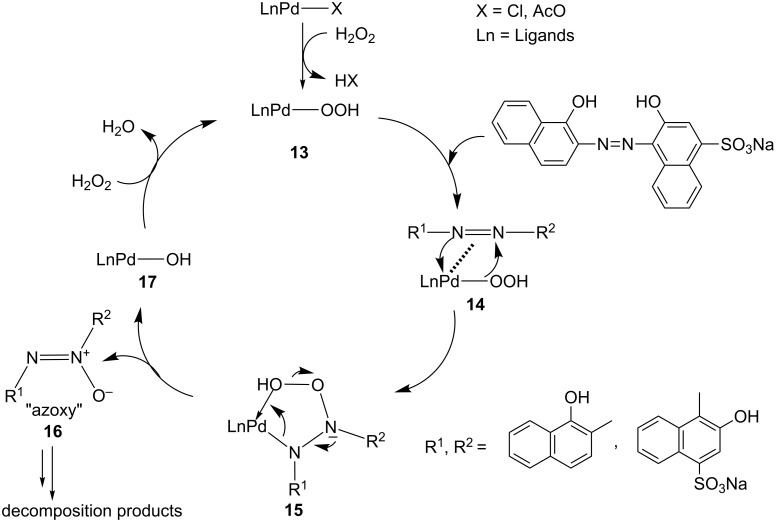
Proposed mechanism of decolorization.

On the evidence of the kinetic studies and the literature data, we propose the mechanistic pathway depicted in Scheme 4. The first step involves the complexation of the azo dye to palladium(II) hydroperoxide **13**, followed by a peroxymetalation of the azo moiety. This then affords the pseudocyclic five membered hydroperoxometallic intermediaite **15** which decomposes to azoxy product **16** and palladium hydroxide complex **17**. The degradation of the azoxy product **16** can afford quinolinones and diazonium salts as it established by many authors [38–40].

## Conclusion

In summary, new palladium-complexes using oxazolines as ligands were synthesized, characterized and tested for the catalytic activity during the degradation of the dye Eriochrome Blue Black B. The variation of the experimental conditions exhibited that the degradation process is contact time, initial dye concentration, temperature and H_2_O_2_ dose-dependent. 84% of target removal of Eriochrome Blue Black B was reached within minutes, under some experimental conditions. These new complexes prove to be active and also to be a reusable catalyst for the decolorization of Erio solutions in the presence of hydrogen peroxide. Further work is ongoing to apply the same strategy for the degradation of other organic pollutants.

## Experimental

### Analytical methods

Unless otherwise noted, all starting materials were obtained from commercial suppliers and used without purification. NMR spectra were recorded on a 300 MHz and 200 MHz Bruker spectrometer. Chemical shifts were reported in ppm relative to the residual solvent peak (7.27 ppm for CHCl_3_) for ^1^H spectra and (77.00 ppm for CDCl_3_) for ^13^C spectra. All chemical shifts were reported as δ values (ppm) relative to internal tetramethylsilane. High resolution mass spectrometry data were recorded on an Autospec Ultima (Waters/Micromass) device with a resolution of 5000 RP at 5%. Thin-layer chromatography (TLC) was carried out on aluminium sheets precoated with silica gel 60 F254. Microwave irradiations were realized using an Anton Paar Monowave 300 apparatus. Microwave heating was performed with a single mode cavity Discover Microwave Synthesizer, producing continuous irradiation with IR temperature control. An ultraviolet–visible spectrophotometer (U-2000 Hitachi), wavelengths of range 200–800 nm and a quartz cell were employed for the absorbance measurements.

### Synthesis of the ligands

The amino acids and (*R*)-2-aminobutan-1-ol were purchased from Sigma-Aldrich. The other α-aminoalcohols were obtained by the reduction of the corresponding amino acids using the method developed by Meyers [41]. The ligands bis- and mono-oxazolines were prepared from the available optically pure α-aminoalcohols (derived from the corresponding amino acids). (*S*)-4-Isopropyl-2-(naphthalen-1-yl)oxazoline (**2**) was isolated in a moderate yield from the condensation of the L-valinol with naphthonitrile under microwave irradiation, while the second ligand 1,2-bis[(*S*)-4-phenyloxazoline]benzene (**7**) was synthesized from L-(α)-(+)-phenylglycinol under the same conditions of the reaction as described by B. Ben Hassine et al. [42]. The third ligand 3-[(4*S*)-4,5-dihydro-4-isopropyl-1,3-oxazol-2-yl]propanenitrile (**11**) was obtained using the reaction of 4-ethoxy-4-iminobutanenitrile monohydrochloride with L-valinol in high yield [43].

### Synthesis of the cyclopalladated complexes

**Synthesis of (*S*)-chloro-[(4-isopropyloxazolinyl)-2-naphthyl](triphenylphosphine)palladium(II) (5):** The complex (**3**) was synthesized using two methods:

**Method A:** A mixture of Pd(OAc)_2_ (50 mg, 0.22 mmol, 1 equiv), AcONa (18.3 mg, 0.22 mmol, 1 equiv) and (*S*)-4-isopropyl-2-(naphthalen-1-yl)oxazoline (**2**, 59 mg, 0.24 mmol, 1.1 equiv) in acetic acid (3.0 mL) was heated in an oil bath at 80 °C for 3.5 h. Complex **3** was isolated in 90% yield.

**Method B:** Pd(OAc)_2_ (50 mg, 0.22 mmol) was added to an acetonitrile solution (3 mL) of oxazoline (**2**) and refluxed for 3 h at 78 °C. The mixture was allowed to cool to rt and filtered through celite. The solvent was evaporated, and the crude product was recrystallized from ether/petroleum ether to obtain **3** (89%).

The metathesis of dimer **3** (0.196 mmol, 1 equiv) with LiCl (18.5 mg, 0.43 mmol, 2.2 equiv) in acetone (7.0 mL) at room temperature for 24 h afforded dimer **4** in 91% yield. PPh_3_ (94.4 mg, 0.36 mmol, 2 equiv) was added to a stirred solution of the dimer **4** (138 mg, 0.18 mmol, 1 equiv) in toluene (10.0 mL). After 12 h, the solvent was evaporated to obtain a pale-yellow solid, which was purified by trituration with petroleum ether or recrystallization from pentane/CH_2_Cl_2_ to afford pure **5** as a yellow powder in 78% yield. [α]_D_ −350 ± 36.9 (*c* 0.02, MeCN); ^1^H NMR (CDCl_3_, 300 MHz) δ 8.03 (dd, *J* = 7.2 Hz, *J* = 1.5 Hz, 1H), 7.80 (dd, *J* = 9.6 Hz, *J* = 1.2 Hz, 1H), 7.73–7.66 (m, 2H), 7.48–7.42, 7.20–7.16 (m, 15H_(PPh3)_), 7.09 (d, 1H), 6.97 (t, 1H), 5.46 (dt, *J* = 9.6 Hz, *J* = 4.8 Hz, CH-N, 1H), 4.58 (m, *J* = 9.6 Hz, *J =* 8.7 Hz, 1H), 4.37 (m, *J* = 8.7 Hz, *J =* 5.4 Hz, 1H), 2.28–2.19 (m, 1H), 0.92 (d, *J* = 6.9 Hz, 3H), 0.71 (d, *J* 6.9 Hz, 3H); ^13^C NMR (CDCl_3_, 75 MHz) δ 163.1, 143.2–123.2 (C(_PPh3_)), 134.8, 132.9, 131.7, 131.1, 129.9, 128.5, 128.0, 127.8, 124.8, 123.9, 70.2, 68.0, 31.3, 18.5, 15.9; ^31^P NMR (MeOD, 75 MHz) δ 36.0 ppm; TOF–MS (ES^+^) for (C_34_H_31_NOPPd): theoretical [M − Cl]^+^: 602.1199; measured [M − Cl]^+^: 602.1201; FTIR (KBr pellets, cm^−1^): 2956.7, 1637.4, 1436.7, 1201.0, 1094.9, 1011.0, 692.3, 513.4.

**Synthesis of dichloro-[1,2-bis((*S*)-4-phenyl-4,5-dihydrooxazol-2-yl)benzene]palladium(II) (8):** Complex **8** was synthesized from 1,2-bis((*S*)-4-phenyl-4,5-dihydrooxazol-2-yl)benzene (**7**) (170 mg, 0.46 mmol, 1.01 equiv) and sodium tetrachloropalladate(II) (134 mg, 0.45 mmol, 1 equiv) in freshly distilled and thoroughly degassed methanol (5 mL). The red solution was allowed to stand for 1 h at room temperature. After filtration, the solid was washed with methanol to afford the expected palladium(II) complex **8** (0.34 mmol) in 75% yield. ^1^H NMR (CDCl_3_, 300 MHz) δ 7.99–7.96 (m, 4H), 7.65–7.58 (m, 5H, H_aromat_), 7.31–7.04 (m, 5H, H_aromat_), 5.88 (dd, *J* = 10.2 Hz, *J* = 5.7 Hz, 1H), 5.06 (t, *J* = 9.3 Hz, 1H), 4.89 (dd, *J* = 9.3 Hz, *J* = 5.7 Hz, 1H), 4.62 (t, *J* = 9.3 Hz, 1H), 4.51 (t, *J =* 9 Hz, 1H), 4.10 (t, *J =* 9 Hz, 1H); ^13^C NMR (CDCl_3_, 75 MHz) δ 162.5, 142.9–124.4, 73.2, 70.5; TOF–MS (ES^+^) for (C_24_H_20_ClN_2_O_2_Pd): theoretical [M − Cl]^+^: 511.0202; measured [M − Cl]^+^: 511.0201.

### Synthesis of the bis(oxazoline) coordinated complexes

**Synthesis of dichlorobis[(4-isopropyl-2-naphthalen-1-yl)oxazoline)]palladium(II) (9) and dichlorobis(4-isopropyl-2-(2-cyanoethyl)oxazoline)palladium(II) (12):** Complexes **9** and **12** were synthesized using the same procedure. A solution of sodium tetrachloropalladate(II) Na_2_PdCl_4_ (0.34 mmol, 1 equiv) in absolute MeOH (3 mL) was added to (0.75 mmol, 2.2 equiv) of the ligand. A yellow precipitate was formed immediately. The mixture was stirred for 24 h at room temperature. After removal of the solvent under reduced pressure, the yellow solid was washed with methanol, and recrystallized from CHCl_3_/hexane. Yields of dichloro[bis(4-isopropyl-2-naphthalen-1-yl)oxazoline]palladium(II) (**9**) and dichloro[bis(4-isopropyl-2-(2-cyanoethyl)oxazoline)]palladium(II) (**12**) are 85% and 68%, respectively.

**Complex 9:** [α]_D_ −113 ± 28 (*c* 0.1, MeCN); ^1^H NMR (CDCl_3_, 300 MHz) δ 8.49 (d, *J* = 6.9 Hz, 1H, H_1_), 8.00–7.97 (m, 2H, H_3,7_), 7.90–7.87 (m, 1H, H_5_), 7.55–7.49 (m, 2H, H_6,7_), 7.43 (bs, 1H, H_2_), 4.58–4.50 (m, 1H, CH-N), 4.46–4.38 (m, 2H, CH_2_-O), 2.62 (m, 1H, CH(CH_3_)_2_), 0.99 (d, *J* = 6.9 Hz, 3H, CH_3_), 0.92 (d, *J* = 6 Hz, 3H, CH_3_); ^13^C NMR (CDCl_3_, 75 MHz) δ 168.9, 133.2, 132.0, 130.7, 130.0, 128.2, 127.1, 126.4, 125.8, 124.6, 124.2, 70.8, 69.3, 30.0, 19.1, 15.3; TOF–MS (ES^+^) for (C_18_H_29_ClN_4_O_2_Pd): theoretical [M − Cl]^+^: 615.1365; measured [M − Cl]^+^: 615.1361; FTIR (KBr pellets, cm^−1^): 2957.8, 1642.2, 1377.8, 1200.4, 1030.8, 776.0, 573.

**Complex 12:** [α]_D_ −5.7 ± 0.5 (*c* 0.94, CHCl_3_); ^1^H NMR (MeOD, 300 MHz) δ 3.64–3.56 (m, 1H), 3.50–3.39 (m, 2H), 2.66–2.45 (m, 1H), 1.83–1.73 (m, 1H), 0.88–0.80 (m, 6H); ^13^C NMR (MeOD, 75 MHz) δ 173.0, 119.4, 64.0, 58.9, 33.1, 30.8, 20.8, 19.6, 14.7; TOF–MS (ES^+^) for (C_18_H_29_Cl_2_N_4_O_2_Pd): theoretical [M + H]^+^: 509.0594; measured [M + H]^+^: 509.0598; FTIR (KBr pellets, cm^−1^): 2963.9, 2250.8, 1635.4, 1545.2, 1260.3, 1018.8.

### Oxidative degradation procedures of dyes

All experiments were carried out in a batch system for a period of time (sufficient to achieve equilibrium) and were uniformly agitated at a speed of 150 rpm. The pH of the solution was adjusted to 7.0 (buffered aqueous solution). 0.2 mg of each catalyst were added to 20 mL of the dye solution (C_0_ = 30 mg/L) followed by the addition of a calculated dose of H_2_O_2_. At the end of each contact time, the content of the flasks was filtered using a Whatman No. 41 filter paper. The concentration of dye in each filtrate was determined at the maximum wavelength (531 nm). Factors affecting the degradation of the dyes such as H_2_O_2_ dose, initial concentration and temperature were studied in the ranges 0–0.7 mL/L, 30–70 mg/L and 22–60 °C, respectively.

## Supporting Information

The electronic Supporting Information includes the X-ray diffraction of the structures of complexes **8** and **9**. The crystallographic data for the structural analysis have been deposited with the Cambridge Crystallographic Data Center, CCDC 1052659-1052660 for **8** and **9**, respectively. Crystallographic data associated with this article can be obtained, free of charge, on application to CCDC, at http://www.ccdc.cam.ac.uk/deposit/.

File 1 CIF file for complex **8**.

File 2 CIF file for complex **9**.

File 3 Experimental procedures, spectroscopic and analytical data, and copies of spectra of the products.
