# Supplementary material for: New palladium–oxazoline complexes: Synthesis and evaluation of the optical properties and the catalytic power during the oxidation of textile dyes
Source: Beilstein J Org Chem. 2015 Jul 15;11:1175–86. doi: 10.3762/bjoc.11.132 (PMC4578361; doi:10.3762/bjoc.11.132)
Supplement: File 3 — Experimental procedures, spectroscopic and analytical data, and copies of spectra of the products. [file Beilstein_J_Org_Chem-11-1175-s003.pdf]

Supporting Information File 3

for

New palladium–oxazoline complexes: Synthesis and evaluation of the optical properties and the catalytic power during the oxidation of textile dyes

Rym Hassani[1], Mahjoub Jabli[1], Yakdhane Kacem[1], Jérôme Marrot[2], Damien Prim[2] and Béchir Ben Hassine*[1]

Address: [1]Laboratoire de Synthèse Organique, Asymétrique et Catalyse Homogène (11URES56). Faculté des sciences de Monastir, Avenue de l'Environnement, 5019 Monastir, Tunisia, Tel: 0021673500279, Fax: 0021673500278 and [2]University of Versailles Saint-Quentin-en-Yvelines, Institut Lavoisier de Versailles, UMR CNRS 8180, 45, avenue des Etats-Unis, 78035 Versailles, France.

*Corresponding author

Email: Béchir Ben Hassine* - bechirbenhassine@yahoo.fr

Experimental procedures, spectroscopic and analytical data, and copies of spectra of the products

Table of contents

1. General methods S2

2. General procedure for synthesis of oxazolines…………………………….................................S2

3. General procedure for synthesis of palladacycle **5**………………………………..........................S4

4. General procedure for synthesis of palladacycle **8**…………………………………….……..............S6

5. General procedure for synthesis of coordinated complexes **9** and **12**……….…..............................S6

4. 1H NMR, 13C NMR, IR-FT and TOF MS$^+$ spectra of the products S9

1. General methods

Unless otherwise noted, all starting materials were obtained from commercial suppliers and used without purification. NMR spectra were recorded on a 300 MHz and 200 MHz Bruker spectrometer. Chemical shifts were reported in ppm relative to the residual solvent peak (7.27 ppm for $CHCl_3$) for ^1H spectra and (77.00 ppm for $CDCl_3$) for ^{13}C spectra. All chemical shifts were reported as δ values (ppm) relative to internal tetramethylsilane. High Resolution Mass spectroscopy data were recorded on an Autospec Ultima (Waters/Micromass) device with a resolution of 5000 RP at 5%. Thin-layer chromatography (TLC) was carried out on aluminium sheets precoated with silica gel 60 F254. Microwave irradiations were realized using an Anton Paar Monowave 300 apparatus. Microwave heating was performed with a single mode cavity Discover Microwave Synthesizer, producing continuous irradiation with IR temperature control. An ultraviolet–visible spectrophotometer (U-2000 Hitachi), wavelengths of range 200–800 nm and a quartz cell were employed for the absorbance measurements.

2. General procedure for synthesis of oxazolines

The ligands bis- and mono-oxazolines were prepared from the available optically pure α-aminoalcohols (derived from the corresponding amino acids).

2.1. Synthesis of (*S*)-4-isopropyl-2-(naphthalen-1-yl)-4,5-dihydro-1,3-oxazole (2)

The synthesis of **2** was described in our previous paper [1]. The naphthonitrile (81 mg, 5.29 mmol, 1 equiv) was mixed with L-valinol (60 mg, 5.82 mmol, 1.1 equiv) in a G4 vial in a Monowave 300 vessel. The resulting mixture was irradiated using the closed vessel mode at 240 °C for 100 min. The reaction mixture was quenched with ethyl acetate and filtered by silica. The filtrate was dried over magnesium sulfate, and concentrated by rota-evaporation under vacuum to give the corresponding (*S*)-4-isopropyl-2-(naphthalen-1-yl)-4,5-dihydro-1,3-oxazole (**2**, 88.5 mg, 3.69 mmol, 70% yield).

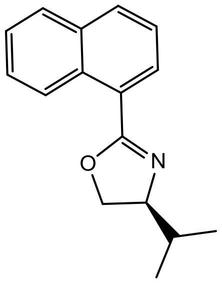

: **(*S*)-4-isopropyl-2-(naphthalen-1-yl)-4,5-dihydro-1,3-oxazole (2):** 70%; Colorless oil; $[\alpha]_D$ = [-63.6 ± 1.9 (*c* = 0.75, $CHCl_3$)]. **^{1}H NMR** ($CDCl_3$, 300 MHz) δ

9.13 (d, 1H; *J* 8.55 Hz), 8.10 (d, 1H; *J* 7.23 Hz), 7.97 (d, 1H; *J* 5.19 Hz), 7.89 (d, 1H; *J* 8.13 Hz), 7.56 (m, 3H), 4.48 (dd, 1H; *J* 7.92 Hz, *J* 9Hz), 4.25 (m, 2H), 1.98 (m, 1H), 1.14 (d, 3H; *J* 6.75 Hz), 1.04 (d, 3H; *J* 9.75 Hz).**^{13}C NMR** ($CDCl_3$, 75 MHz) δ 163.3, 133.7, 131.7, 131.2, 128.9, 128.4, 127.2, 126.4, 126.0, 124.7, 124.6, 73.3, 69.3, 32.9, 19.0, 18.3. TOFMS ES^+ for $C_{14}H_{13}N_3O$ theoretical $[M+H]^+$: 240.1388; measured $[M+H]^+$:240.1392. **IR-TF** (KBr pellets, cm^{-1}) ν 777.7, 1025.3, 1123.1, 1359.2, 1465.2, 1590.5, 1652.6, 2958.5, 3050.2.

2.2. Synthesis of 1,2-bis[(*S*)-4-phenyloxazoline]benzene (7)

The 1,2-bis((*S*)-4-isopropyloxazoline)benzene **7** was synthesized from the L-(+)-α-phenylglycinol under the same conditions of the reaction as described in our previous paper [1].
A G4 vial in a Monowave 300 vessel was charged with phthalonitrile (25.5 mg, 0.198 mmol, 1 equiv) was mixed with L-(+)-α-phenylglycinol (60 mg, 0.437 mmol, 2.2 equiv). The resulting mixture was irradiated using the closed vessel mode at 150 °C for 60 min. The reaction mixture was quenched with ethyl acetate and filtered by silica. The filtrate was dried over magnesium sulfate, and concentrated by rota-evaporation under vacuum to give the corresponding 1,2-bis[(*S*)-4-phenyl-4,5-dihydro-1,3-oxazol-2-yl]benzene (**7**) (98% yield) as a colorless oil.

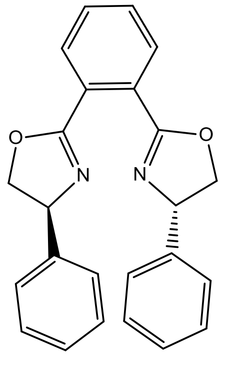

: **1,2-bis[(*S*)-4-phenyl-4,5-dihydro-1,3-oxazol-2-yl]benzene (7):** 98%; Yellow oil; **^{1}H NMR** ($CDCl_3$, 300 MHz) δ 7.76 (dd, 1H; *J* 6 Hz, *J* 3.6 Hz), 7,47 (dd, 1H; *J* 6 Hz, *J* 3.3 Hz), 4.34-4.43 (m, 1H), 4.05-4.14 (m, 2H), 0.96 (d, 3H; *J* 6.6 Hz). **^{13}C NMR** ($CDCl_3$, 75 MHz) δ 164.9, 142.2-126.9 (C_{aromat}), 75.2, 70.5.

2.3. Synthesis of 3-[(4*S*)-4-isopropyl-4,5-dihydro-1,3-oxazol-2-yl]propanenitrile (11):

The third ligand 3-[(4*S*)-4-isopropyl-4,5-dihydro-1,3-oxazol-2-yl]propanenitrile (**11**) was obtained using the reaction of iminoether with the L-valinol [2]. In a Schlenk tube equipped

with a magnetic bar, the monohydrochloride of the 4-ethoxy-4-iminobutanenitrile (1.58 g, 9.69 mmol) and the L-valinol (1 g, 9.69 mmol) were introduced respectively in 50 mL of anhydrous methylene chloride. Then, the mixture was heated at reflux for 10 hours under an inert atmosphere. After the removal of NH_4Cl by filtration, the organic phase was evaporated and the obtained residue was chromatographed on silica gel to afford 89% of the compound **11.**

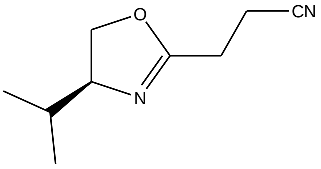

: **3-[(4*S*)-4-isopropyl-4,5-dihydro-1,3-oxazol-2-yl]propanenitrile (11)**: 89%; Yellow oil; $[\alpha]_D$ = [-20; *c* 1, $CHCl_3$]. **^{1}H NMR**($CDCl_3$, 300 MHz) δ 0.78 (d, 3H; *J* 6.8 Hz), 0.85 (d, 3H; *J* 7 Hz), 1.12-1.22 (m, 1H), 1.57-1.73 (m, 1H), 2.47-2.68 (m,4H), 3.75-3.94 (m,1H), 4.04-4.22 (m,1H).**^{13}C NMR** ($CDCl_3$,75 MHz) δ 14.23 (CH_2), 18.10 (CH_3), 18.67, 24.24, 32.56, 70.63, 72.13, 118.68, 163.66. **TOFMS ES$^+$** for $C_9H_{14}N_2O$ theoretical $[M+H]^+$:167.1184; measured $[M+H]^+$: 167.1180.

3. General procedure for synthesis of palladacycle 5

Complex **3** was synthesized using two methods according to the procedure adopted from ref. [3]:

Method A: A mixture of $Pd(OAc)_2$ (50mg, 0.22 mmol, 1equiv), AcONa (18.3mg, 0.22 mmol, 1eq) and oxazoline **2** (59 mg, 0.24 mmol, 1.1 equiv) in acetic acid (3.0 mL) was heated in an oil bath at 80 °C for 3.5 h. Complex **3** was isolated in 90% yield.

Method B: $Pd(OAc)_2$ (50 mg, 0.22 mmol) was added to an acetonitrile solution (3 mL) of oxazoline **2** and refluxed for 3 h at 78 °C. The mixture was allowed to cool to rt and filtered through celite. The solvent was evaporated, and the crude product was recrystallized from ether/petroleum ether to obtain **3** (89%).

The metathesis of dimer **3** (0.196 mmol, 1 equiv) with LiCl (18.5 mg, 0.43 mmol, 2.2 equiv) in acetone (7.0 mL) at room temperature for 24 h afforded the dimer **4** in 91% yield. PPh_3 (94.4 mg, 0.36 mmol, 2equiv) was added to a stirred solution of the dimer **4** (138 mg, 0.18 mmol, 1 equiv) in toluene (10.0 mL). After 12 h, the solvent was evaporated to obtain a pale-yellow solid, which was purified by trituration with petroleum ether or recrystallization from pentane/CH_2Cl_2 to afford the pure **5** as a yellow powder in 78% yield.

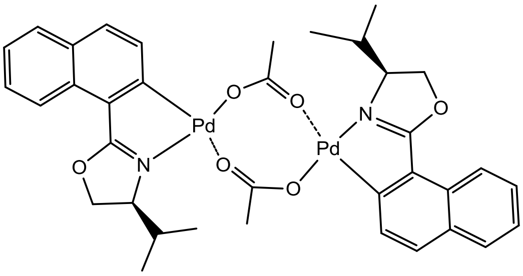

: **(*S*,*S*)-di-µ-acetatobis-{2-[2-(4-isopropyl)-oxazolinyl]naphtyl-*C*,*N*}dipalladium(II) (3):** 90%; orange solid**; [α]$_D$=** - 64±5 [*c* 0.85 g/100mL, $CHCl_3$]**; ^{1}H NMR** ($CDCl_3$, 300 MHz) δ 7.96 (dd, 2H, *J* 11.7 Hz, *J* 2.1 Hz), 7.74 (d, 2H; *J* 11.1 Hz), 7.61 (d, 2H; J 11.4 Hz), 7.48-7.15 (m, 6H), 4.87-4.78 (m, 2H), 4.66-4.38 (m, 4H), 2.75-2.45 (m, 2H), 2.14 (s, 6H), 1.07-0.79 (m, 12H).

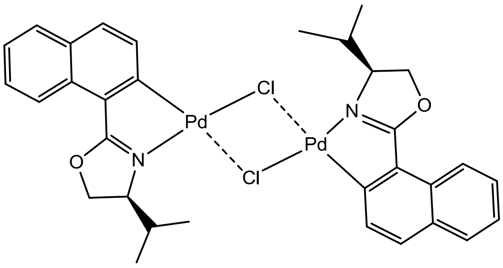

: **(*S*,*S*)-di-µ-chlorobis-{2-[2-(4-isopropyl)-oxazolinyl]naphtyl-C,N}dipalladium(II) (4):** 91%; orange solid**; [α]$_D$=** +83±22 [*c* 1, $CHCl_3$]**. ^{1}H NMR** ($CDCl_3$, 300 MHz) δ 8.09-7.19 (m, $12H_{naphthyl}$), 4.85-4.76 (m, 2H), 4.66-4.41 (m, 4H), 2.66-2.57 (m, 2H), 1.12-0.75 (m, 12H). **^{13}C NMR** ($CDCl_3$, 75 MHz) δ 167.3, 133.7, 132.9, 132.0, 131.3, 130.0, 128.4, 128.2, 126.8, 124.9, 124.0, 71.3, 69.1, 31.2, 19.6, 15.6. **TOFMS ES$^+$** for ($C_{32}H_{32}N_2O_2$ Cl^{102}Pd^{104}Pd): theoretical [M-Cl]$^+$: 717.0249; measured [M-Cl]$^+$: 717.0249. **IR-FT** (KBr pellets, cm^{-1}) ν 2954.8, 1641.6, 1355.3, 1210.0, 1012.9, 766.2, 510.9.

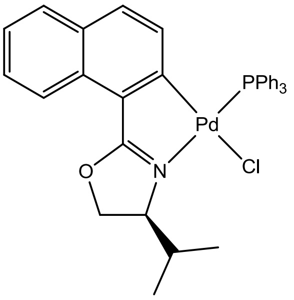

: **(*S*)-Chloro-[(4-isopropyl-oxazolinyl)-2-naphthyl]-(triphenylphosphine)palladium(II) (5): [α]$_D$** = -350±36.9 (*c* 0.02, MeCN). **^{1}H NMR** ($CDCl_3$, 300 MHz) δ 8.03 (dd, 1H, *J* 7.2 Hz, *J* 1.5 Hz), 7.80 (dd, 1H; *J* 9.6 Hz, *J* 1.2 Hz), 7.73-7.66 (m, 2H), 7.48-7.42 et 7.20-7.16 (m, $15H_{(PPh3)}$), 7.09 (d, 1H), 6.97 (t, 1H), 5.46 (dt,

1H; *J* 9.6 Hz, *J* 4.8 Hz, CH-N), 4.58 (m, 1H; *J* 9.6 Hz, *J* 8.7 Hz), 4.37 (m, 1H; *J* 8.7 Hz, *J* 5.4 Hz), 2.28-2.19 (m, 1H), 0.92 (d, 3H, *J* 6.9 Hz), 0.71 (d, 3H, *J* 6.9 Hz). **^{13}C NMR** ($CDCl_3$, 75 MHz) δ 163.1, 143.2-123.2 ($C_{(PPh3)}$), 134.8, 132.9, 131.7, 131.1, 129.9, 128.5, 128.0, 127.8, 124.8, 123.9, 70.2, 68.0, 31.3, 18.5, 15.9. ^{31}P NMR (MeOD, 75 MHz, δ (ppm)): 36.0 ppm. **TOFMS ES$^+$** for ($C_{34}H_{31}NOPPd$): theoretical $[M-Cl]^+$: 602.1199; measured $[M-Cl]^+$: 602.1201. **IR-FT** (KBr pellets, cm^{-1})**:** 2956.7, 1637.4, 1436.7, 1201.0, 1094.9, 1011.0, 692.3, 513.4.

4. General procedure for synthesis of palladacycle 8

Complex **8** was synthesized from 1,2-bis[(*S*)-4-phenyl-4,5-dihydrooxazol-2-yl]benzene (**7**) (170 mg, 0.46 mmol, 1.01 equiv) and sodium tetrachloropalladate(II) (134 mg, 0.45 mmol, 1 equiv) in freshly distilled and thoroughly degassed methanol (5 mL). The red solution was allowed to stand for 1 h at room temperature. After filtration, the solid was washed with methanol to afford the expected palladium(II) complex **8** (0.34 mmol) in 75% yield.

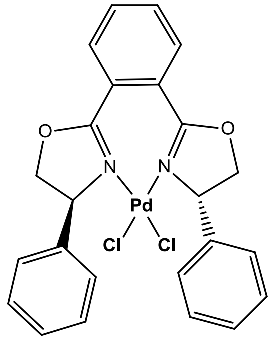

: **Dichloro-{1,2-bis[(*S*)-4-phenyl-4,5-dihydrooxazol-2-yl]benzene}-palladium (8):** 75%; Yellow crystal; **^{1}H NMR** ($CDCl_3$, 300 MHz) δ 7.99-7.96 (m, 4H), 7.65-7.58 (m, 5H, H_{aromat}), 7.31-7.04 (m, 5H; H_{aromat}), 5.88 (dd, 1H*;* *J* 10.2 Hz, *J* 5.7 Hz), 5.06 (t, 1H; *J* 9.3 Hz), 4.89 (dd, 1H*;* *J* 9.3 Hz, *J* 5.7 Hz), 4.62 (t, 1H; *J* 9.3 Hz), 4.51 (t, 1H; *J* 9 Hz), 4.10 (t, 1H; *J* 9 Hz). **^{13}C NMR** ($CDCl_3$, 75 MHz) δ 162.5, 142.9-124.4, 73.2, 70.5. **TOFMS ES$^+$** for ($C_{24}H_{20}ClN_2O_2Pd$): theoretical $[M-Cl]^+$: 511.0202; measured $[M-Cl]^+$: 511.0201.

5. General procedure for synthesis of coordinated complexes 9 and 12

Complexes **9** and **12** were synthesized using the same procedure. A solution of sodium tetrachloropalladate(II) Na_2PdCl_4 (0.34 mmol, 1equiv) in absolute MeOH (3 mL) was added

to (0.75 mmol, 2.2 equiv) of the ligand. A yellow precipitate was formed immediately. The mixture was stirred for 24 h at room temperature. After removal of the solvent under reduced pressure, the yellow solid was washed with methanol, and recrystallized from $CHCl_3$/hexane. Yields of dichlorobis[(4-isopropyl-2-naphthalen-1-yl)oxazoline]palladium(II) **9** and dichlorobis(4-isopropyl-2-cyanoethyl-oxazoline)palladium(II) **12** are 85% and 68% respectively.

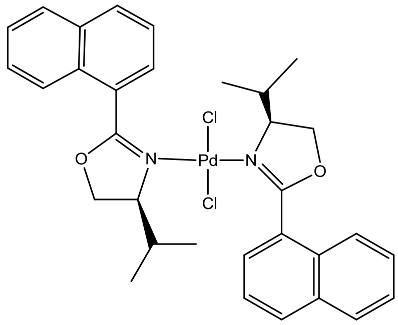

: **Dichlorobis[(4-isopropyl-2-naphthalen-1-yl)oxazoline] palladium (II) (9):** $[\alpha]_D$= -113±28 (*c* 0.1, MeCN). **^{1}H NMR** ($CDCl_3$, 300 MHz) δ 8.49 (d, 1H, *J* 6.9 Hz, H_1), 8.00-7.97 (m, 2H; $H_{3,7}$), 7.90-7.87 (m, 1H; H_5), 7.55-7.49 (m, 2H; $H_{6,7}$), 7.43 (bs, 1H; H_2), 4.58-4.50 (m, 1H; CH-N), 4.46-4.38 (m, 2H; CH_2-O), 2.62 (m, 1H, $CH(CH_3)_2$), 0.99 (d, 3H, *J* 6.9 Hz, CH_3), 0.92 (d, 3H, *J* 6 Hz, CH_3). **^{13}C NMR** ($CDCl_3$, 75 MHz) δ 168.9, 133.2, 132.0, 130.7, 130.0, 128.2, 127.1, 126.4, 125.8, 124.6, 124.2, 70.8, 69.3, 30.0, 19.1, 15.3. **TOFMS ES$^+$** for ($C_{18}H_{29}ClN_4O_2Pd$): theoretical $[M-Cl]^+$: 615.1365; measured $[M-Cl]^+$: 615.1361. IR-FT (KBr pellets, cm^{-1}) ν 2957.8, 1642.2, 1377.8, 1200.4, 1030.8, 776.0, 573.

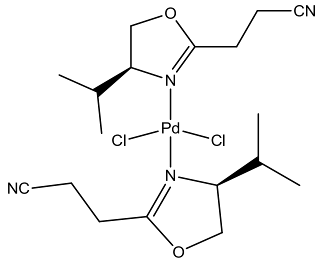

: **Dichlorobis(4-isopropyl-2-cyanoethyl-oxazoline)palladium(II) (12): $[\alpha]_D$=** -5.7±0.5 (*c* 0.94, $CHCl_3$). **^{1}H NMR** (MeOD, 300 MHz) δ 3.64-3.56 (m, 1H), 3.50-3.39 (m, 2H), 2.66-2.45 (m, 1H), 1.83-1.73 (m, 1H), 0.88-0.80 (m, 6H). **^{13}C NMR** (MeOD, 75 MHz) δ 173.0, 119.4, 64.0, 58.9, 33.1, 30.8, 20.8, 19.6, 14.7. **TOFMS ES$^+$** for ($C_{18}H_{29}Cl_2N_4O_2Pd$): theoretical $[M+H]^+$: 509.0594; measured $[M+H]^+$: 509.0598. **IR-FT** (KBr pellets, cm^{-1}): 2963.9, 2250.8, 1635.4, 1545.2, 1260.3, 1018.8.

References:

[1]: Hassani, R.; Requet, A.; Marque, S.; Gaucher, A.; Prim, D.; Kacem, Y.; Ben Hassine, B. *Tetrahedron : Asymmetry* **2014**, *25*, 1275–1279.

[2]: Hassani, R.; Kacem, K.; Ben Mansour, H.; Ben Ammar, H.; Ben Hassine, B. *Chem. Bio. Inter*. **2014**, *217*, 41–48.

[3]: Dunina, V.V.; Zalevskaya, O.A.; Potapov, V.M. *Russ. Chem. Rev*. **1988**, 57, 434.

[4]: Mawo, R.Y.; Mustakim, S.; Young Jr., V.G.; Hoffmann, M.R.; Smoliakova, I.P. *Organometallics* **2007**, *26*, 1801.

4. ^{1}H NMR, ^{13}C NMR, IR-FT and TOF MS$^+$ spectra of the products

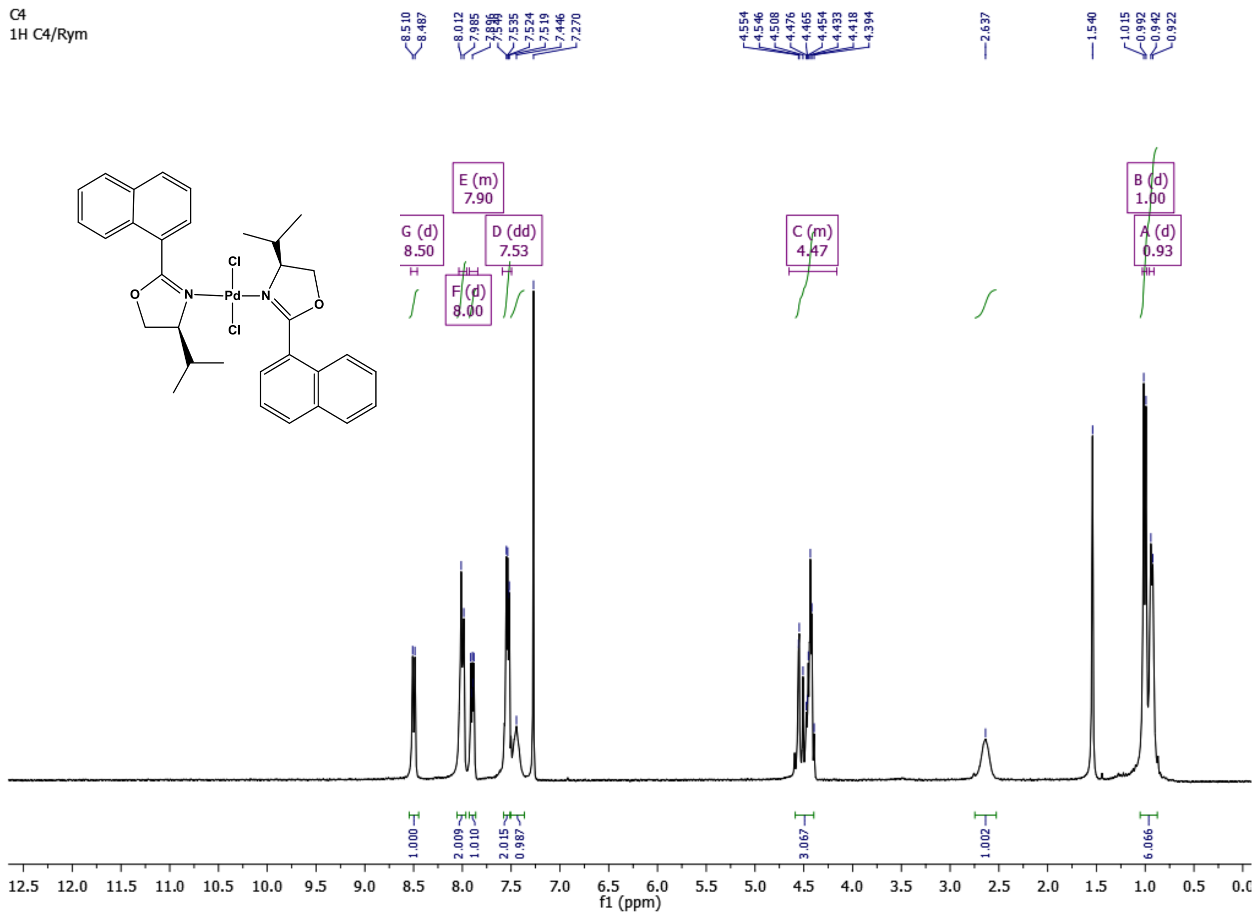

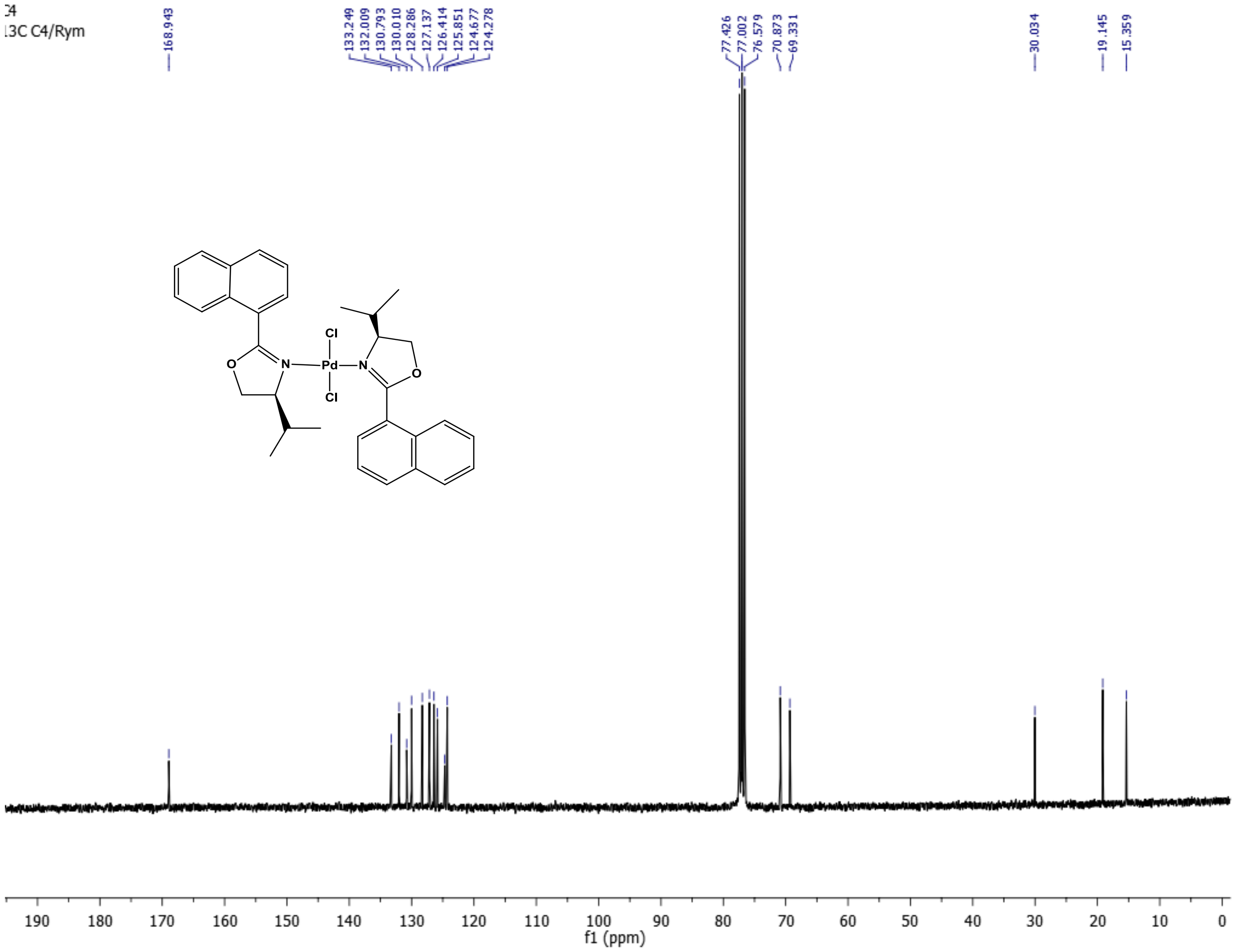
C4
13C C4/Rym
168.943
133.249
132.009
130.793
130.010
128.286
127.137
126.414
125.851
124.677
124.278
77.426
77.002
76.579
70.873
69.331
30.034
19.145
15.359
Cl
Pd
N
O
Cl
190 180 170 160 150 140 130 120 110 100 90 80 70 60 50 40 30 20 10 0
f1 (ppm)

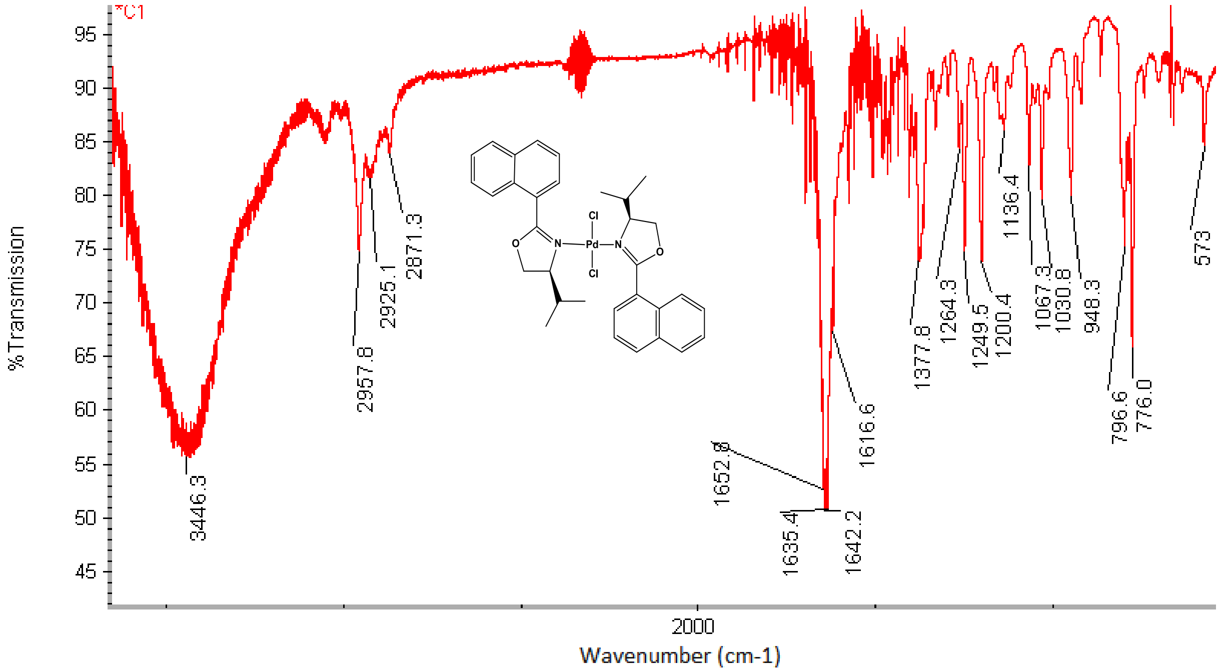
%Transmission
95 90 85 80 75 70 65 60 55 50 45
3446.3
2957.8
2925.1
2871.3
1652.8
1635.4
1642.2
1616.6
1377.8
1264.3
1249.5
1200.4
1136.4
1067.3
1030.8
948.3
796.6
776.0
573
2000
Wavenumber (cm-1)

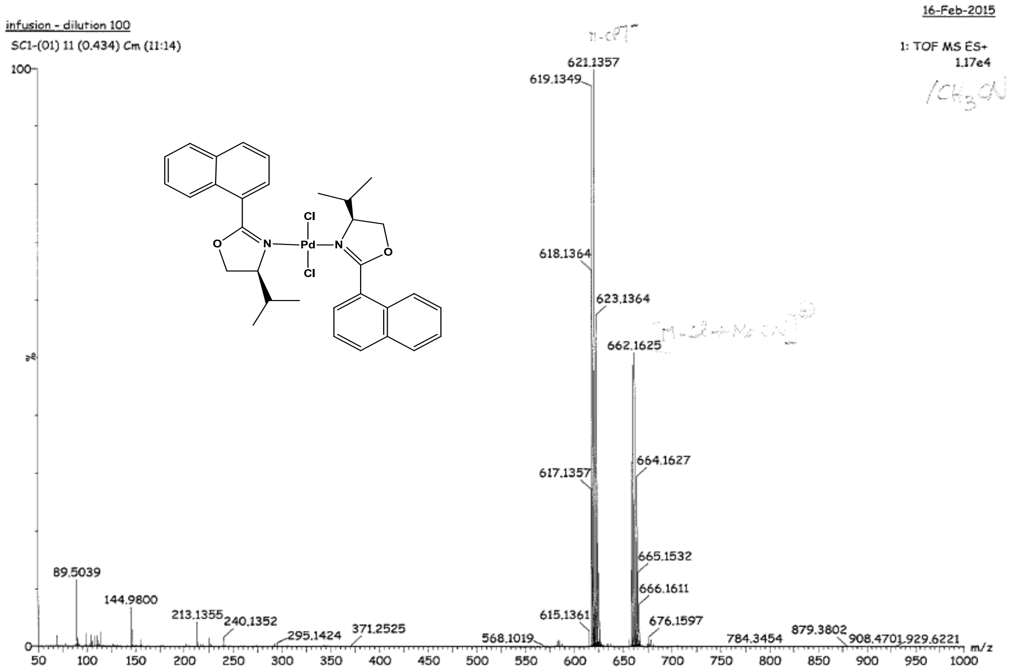

Elemental Composition Report

Page 1

Single Mass Analysis

Tolerance = 2.0 PPM / DBE: min = -1.5, max = 500.0
Element prediction: Off
Number of isotope peaks used for i-FIT = 3

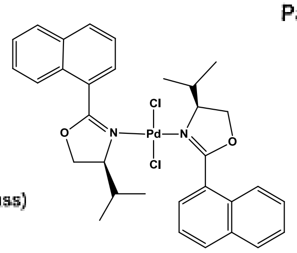

Monoisotopic Mass, Even Electron Ions
1323 formula(e) evaluated with 5 results within limits (all results (up to 1000) for each mass)
Elements Used:
C: 0-40 H: 0-40 N: 0-5 O: 0-5 Cl: 0-5 102Pd: 0-1

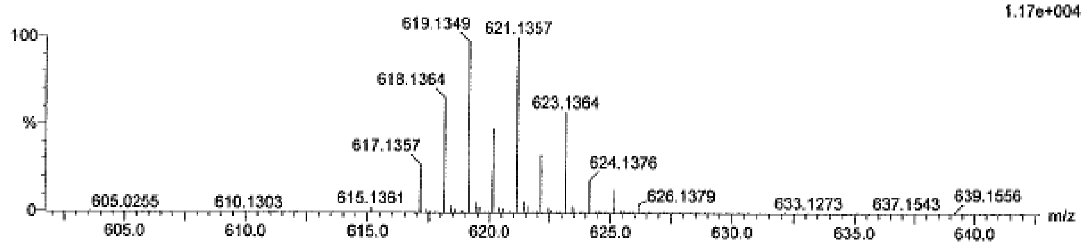

Minimum:				-1.5			
Maximum:		10.0	2.0	500.0			
Mass	Calc. Mass	mDa	PPM	DBE	i-FIT	i-FIT (Norm)	Formula
615.1361	615.1365	-0.4	-0.7	16.5	106.1	1.1	C32 H34 N2 O2 Cl 102Pd

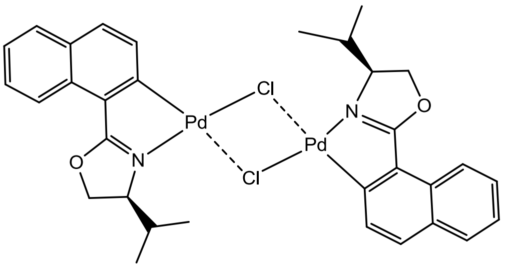

Elemental Composition Report

Single Mass Analysis
Tolerance = 5.0 PPM / DBE: min = -1.5, max = 500.0
Element prediction: Off
Number of isotope peaks used for i-FIT = 3

Monoisotopic Mass, Even Electron Ions
141145 formula(e) evaluated with 171 results within limits (all results (up to 1000) for each mass)
Elements Used:
C: 0-40 H: 0-40 N: 0-5 O: 0-5 Cl: 0-5 102Pd: 0-2 104Pd: 0-2 105Pd: 0-2 106Pd: 0-2 108Pd: 0-2
110Pd: 0-2

Sylvain MARQUE 16-Feb-2015
Infusion - dilution 100
SD-(01) 20 (0.761) Cm (20) 1: TOF MS ES+
7.61e+003

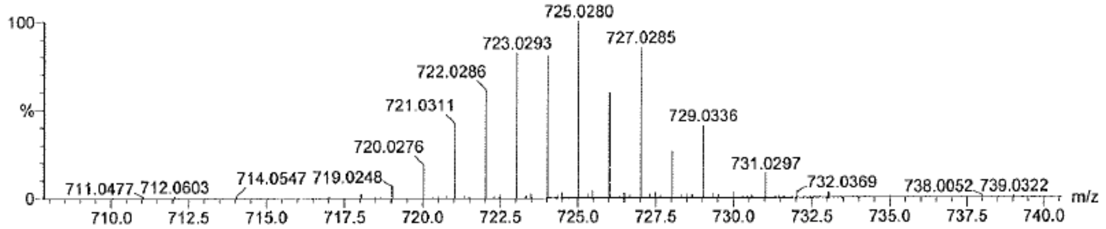

Minimum:				-1.5			
Maximum:		10.0	5.0	500.0			
Mass	Calc. Mass	mDa	PPM	DBE	i-FIT	i-FIT (Norm)	Formula
717.0249	717.0249	0.0	0.0	10.5	51.7	7.5	C28 H39 N2 102Pd 104Pd 108Pd
	717.0249	0.0	0.0	17.5	49.6	5.4	C32 H32 N2 O2 Cl 102Pd 104Pd

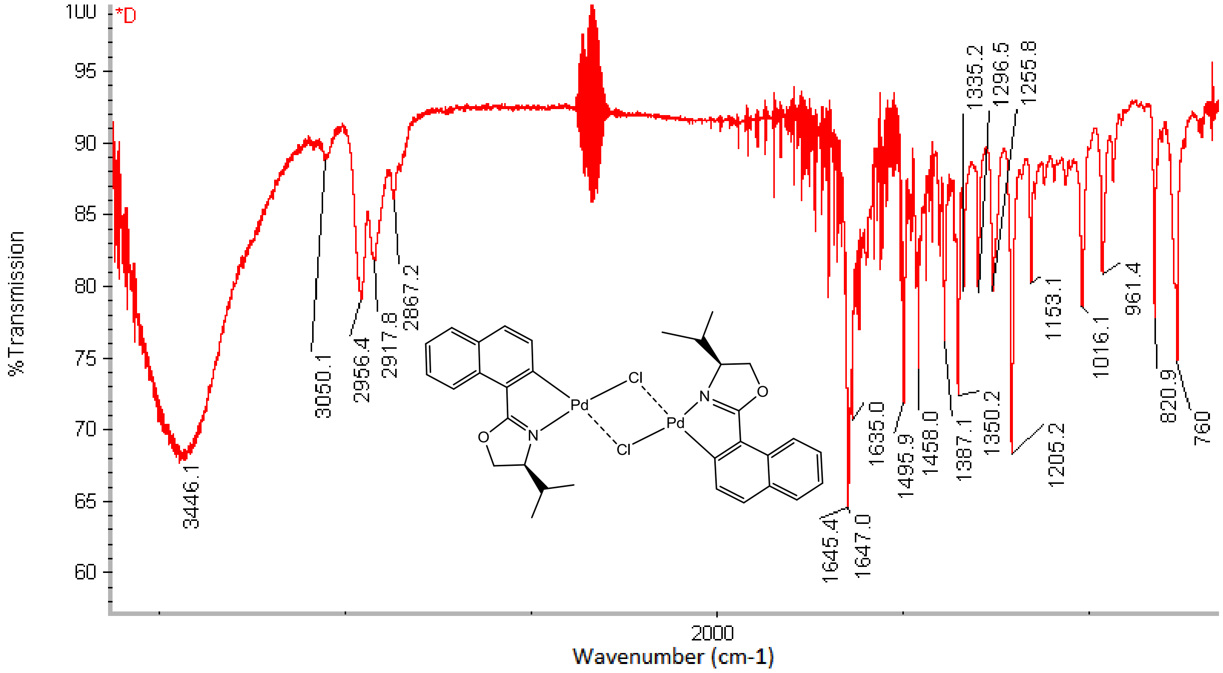

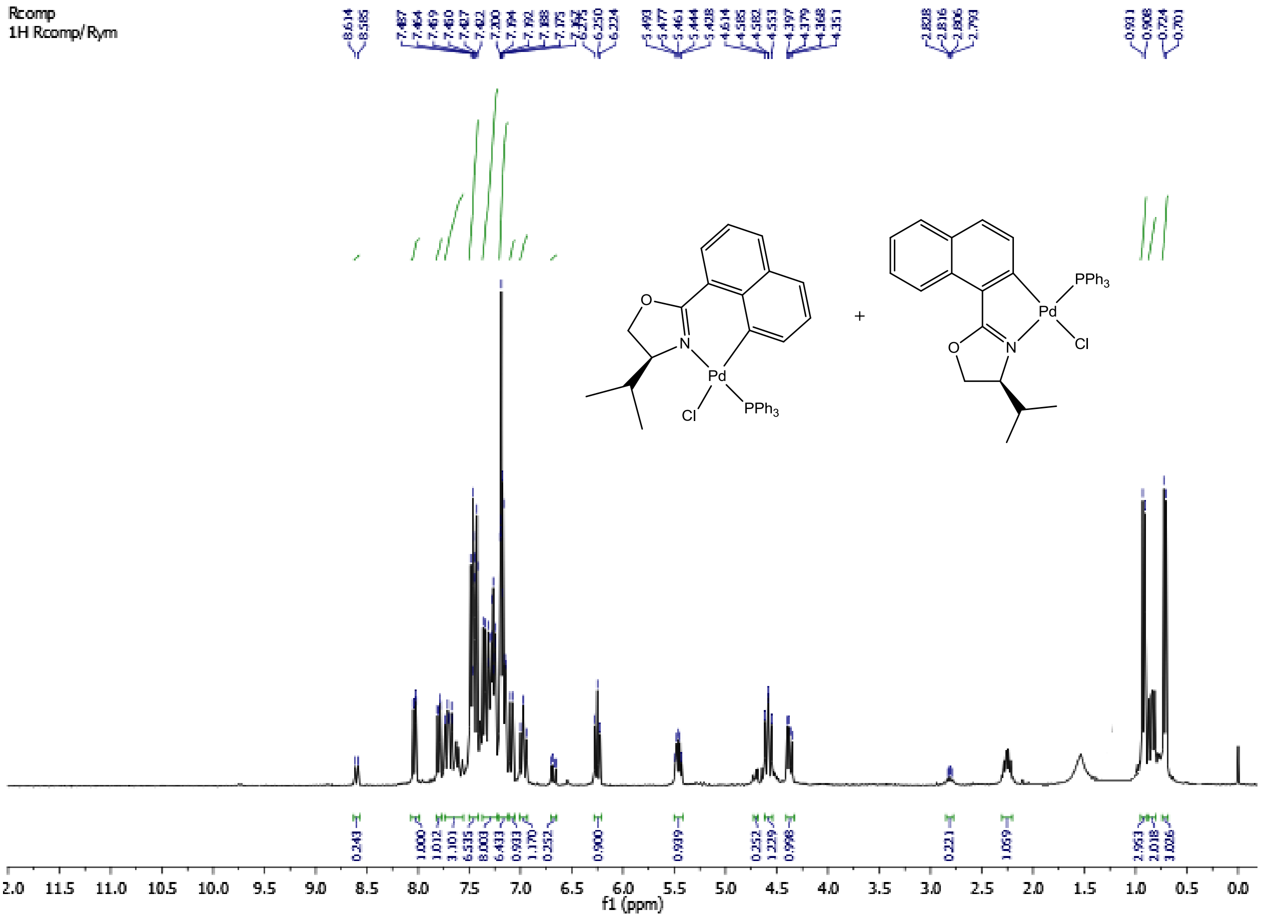
Rcomp
1H Rcomp/Rym
O
N
Pd
Cl
PPh3
+
f1 (ppm)

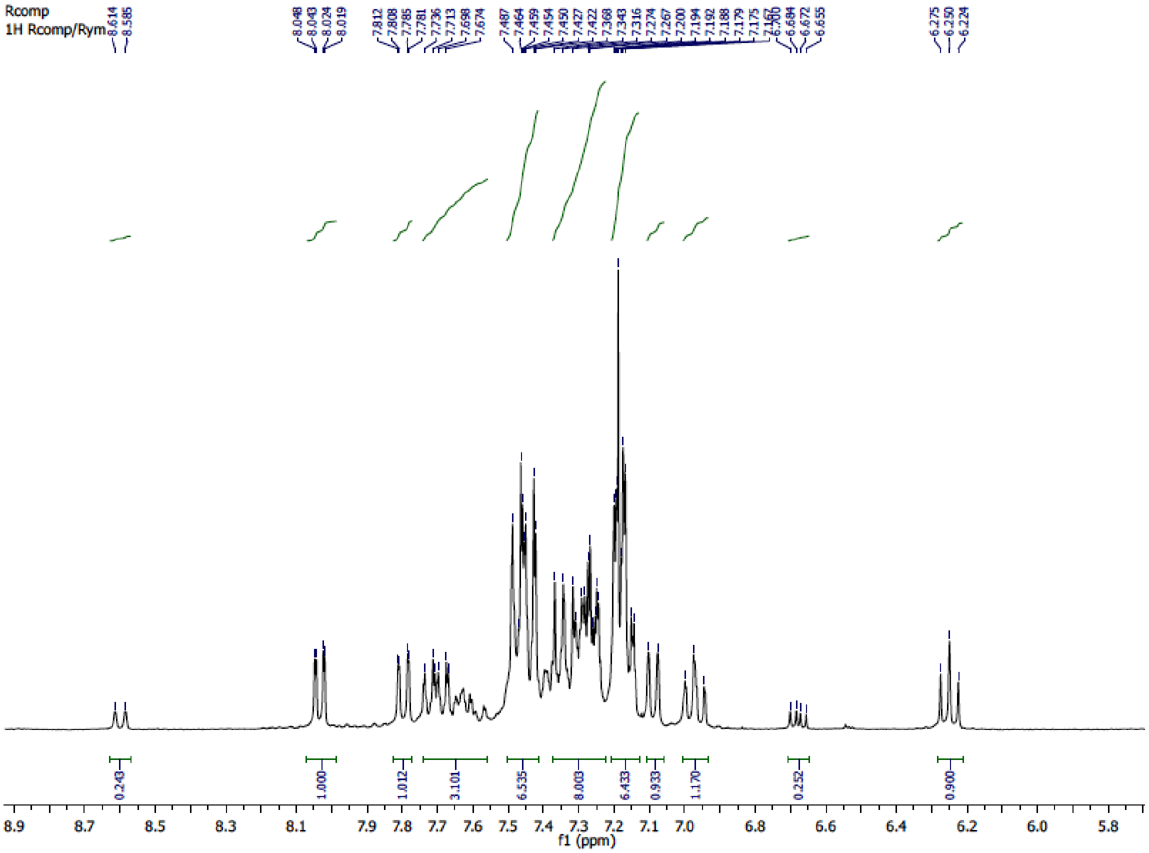
Rcomp
1H Rcomp/Rym
f1 (ppm)

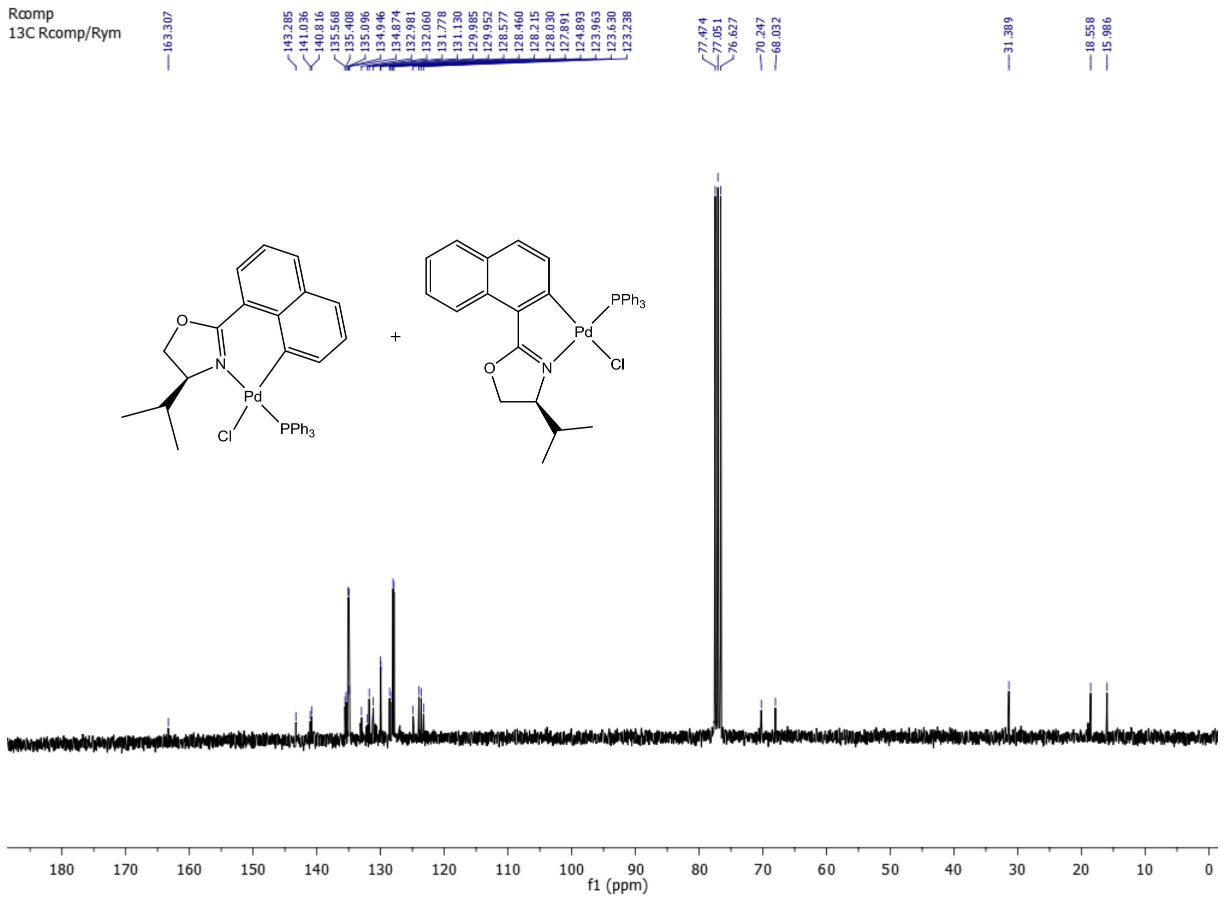

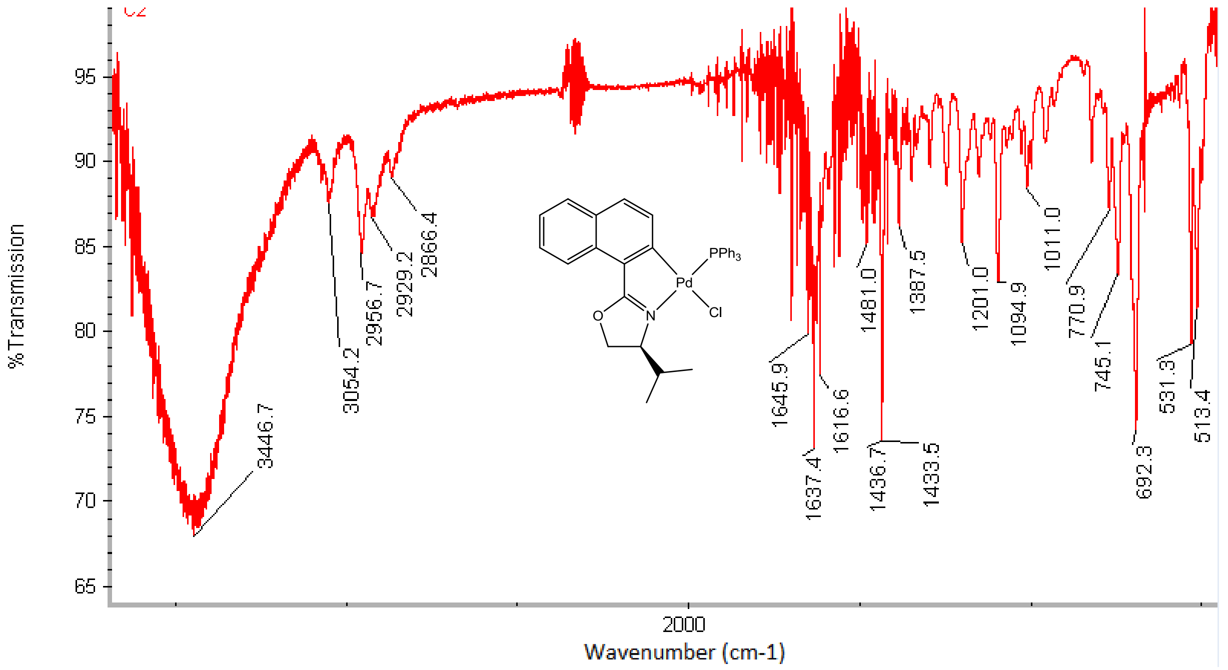

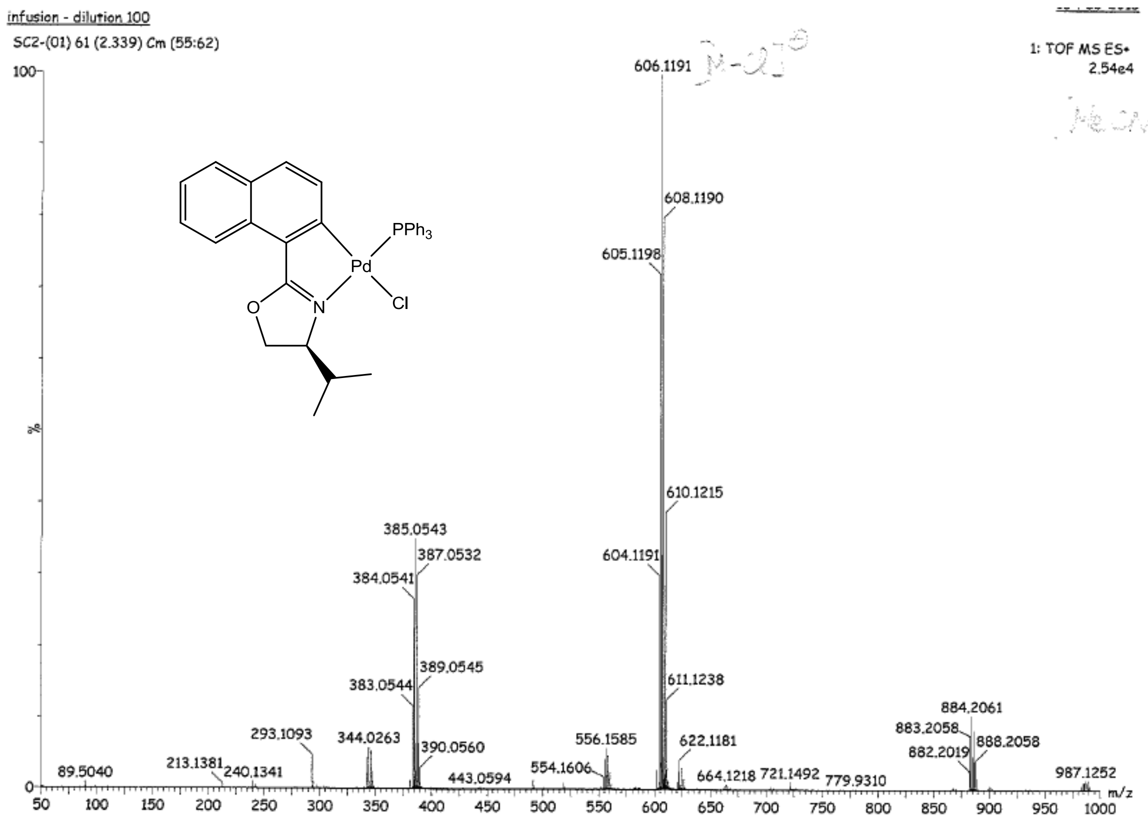

Elemental Composition Report

Page 1 of 1

Single Mass Analysis

Tolerance = 2.0 PPM / DBE: min = -1.5, max = 500.0
Element prediction: Off
Number of isotope peaks used for i-FIT = 3

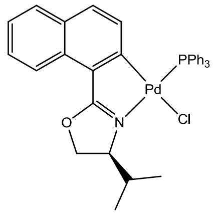

Monoisotopic Mass, Even Electron Ions
5751 formula(e) evaluated with 16 results within limits (all results (up to 1000) for each mas
Elements Used:
C: 0-40 H: 0-40 N: 0-5 O: 0-5 Cl: 0-5 102Pd: 0-1 P: 0-3

16-Feb-2015

infusion - dilution 100
SC2-(01) 61 (2.339) Cm (55:62)

OF MS ES+
2.54e+004

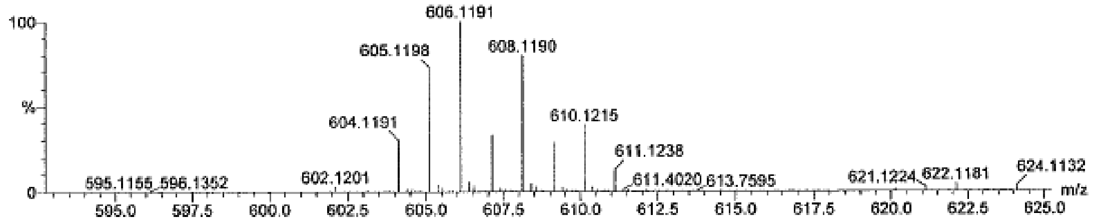

Minimum:				-1.5			
Maximum:		10.0	2.0	500.0			
Mass	Calc. Mass	mDa	PPM	DBE	i-FIT	i-FIT (Norm)	Formula
602.1201	602.1201	0.0	0.0	20.5	152.9	2.1	C35 H31 N Cl 102Pd
	602.1199	0.2	0.3	20.5	159.6	8.8	C34 H31 N O 102Pd P

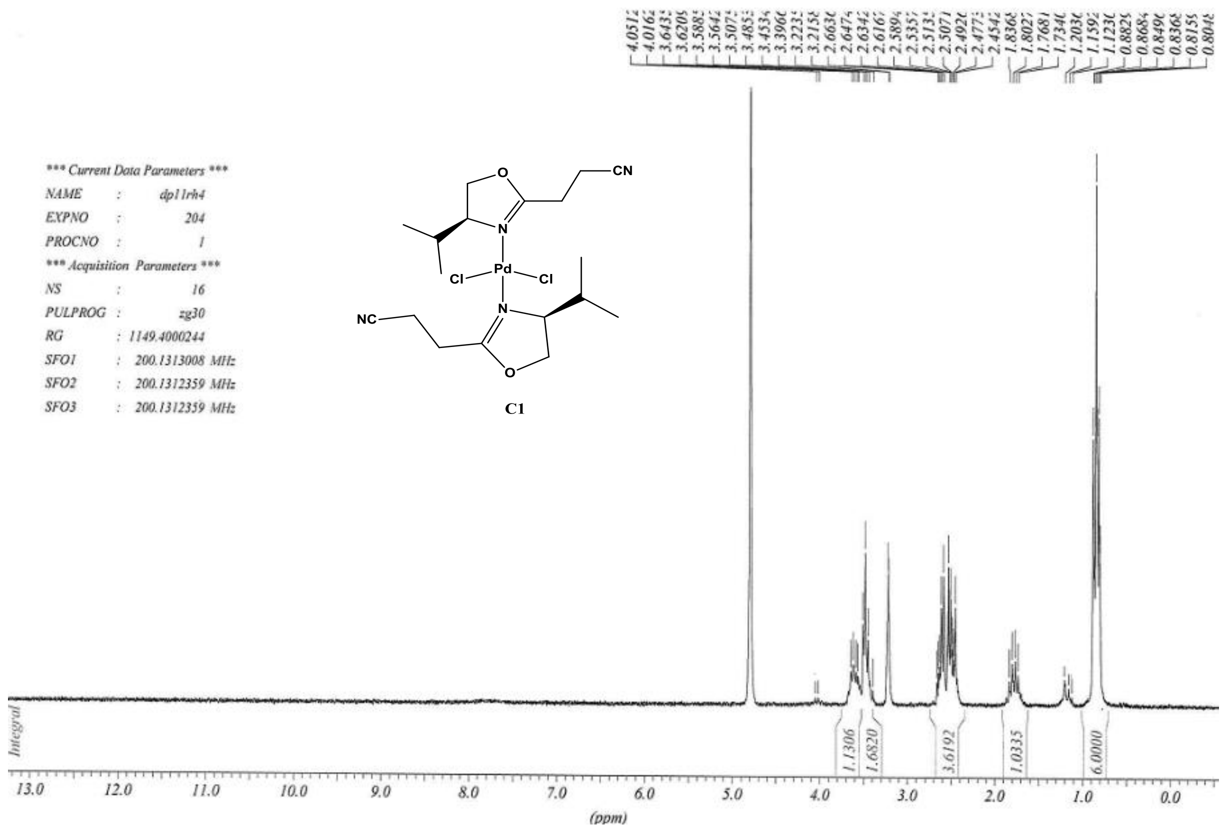
*** Current Data Parameters ***
NAME : dp11rh4
EXPNO : 204
PROCNO : 1
*** Acquisition Parameters ***
NS : 16
PULPROG : zg30
RG : 1149.4000244
SFO1 : 200.1313008 MHz
SFO2 : 200.1312359 MHz
SFO3 : 200.1312359 MHz
O
CN
N
Pd
Cl
Cl
N
NC
O
C1
Integral
1.1306
1.6820
3.6192
1.0335
6.0000
13.0 12.0 11.0 10.0 9.0 8.0 7.0 6.0 5.0 4.0 3.0 2.0 1.0 0.0
(ppm)

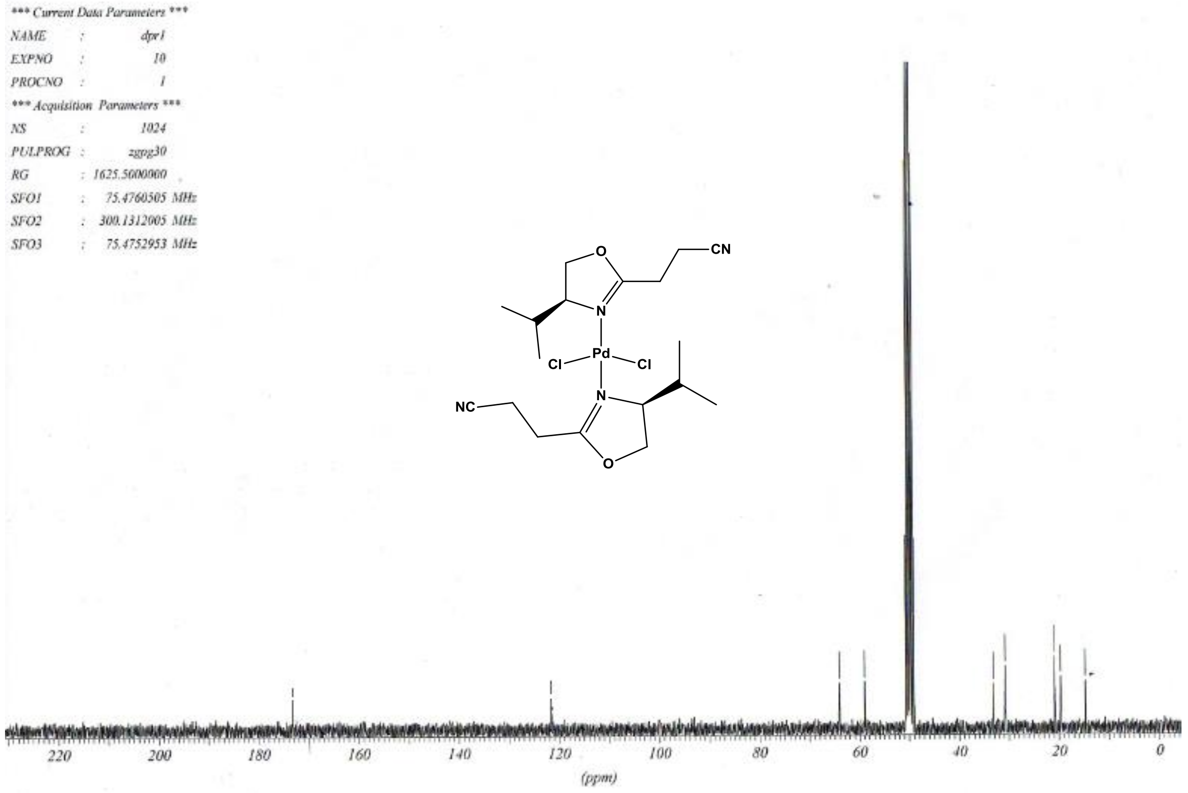
*** Current Data Parameters ***
NAME : dpr1
EXPNO : 10
PROCNO : 1
*** Acquisition Parameters ***
NS : 1024
PULPROG : zgpg30
RG : 1625.5600000
SFO1 : 75.4760505 MHz
SFO2 : 300.1312005 MHz
SFO3 : 75.4752953 MHz
O
CN
N
Pd
Cl
Cl
N
NC
O
220 200 180 160 140 120 100 80 60 40 20 0
(ppm)

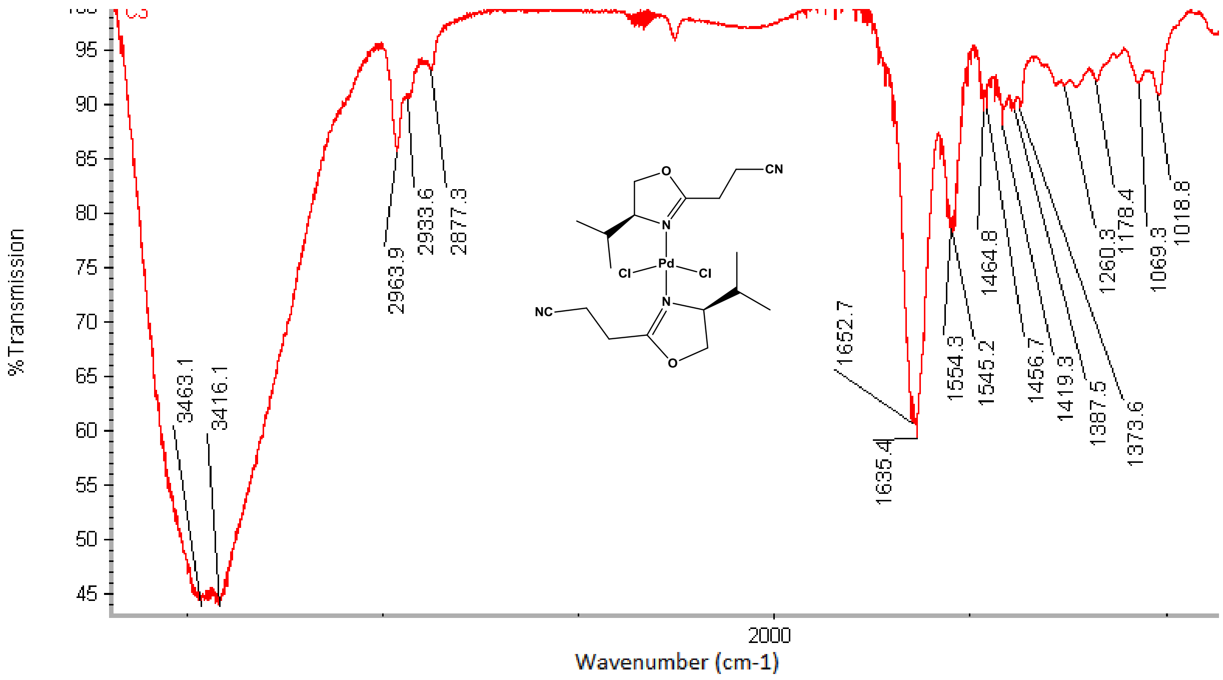
% Transmission
Wavenumber (cm-1)
2000
95
90
85
80
75
70
65
60
55
50
45
3463.1
3416.1
2963.9
2933.6
2877.3
1652.7
1635.4
1554.3
1545.2
1464.8
1456.7
1419.3
1387.5
1373.6
1260.3
1178.4
1069.3
1018.8
CN
NC
Pd
Cl
Cl

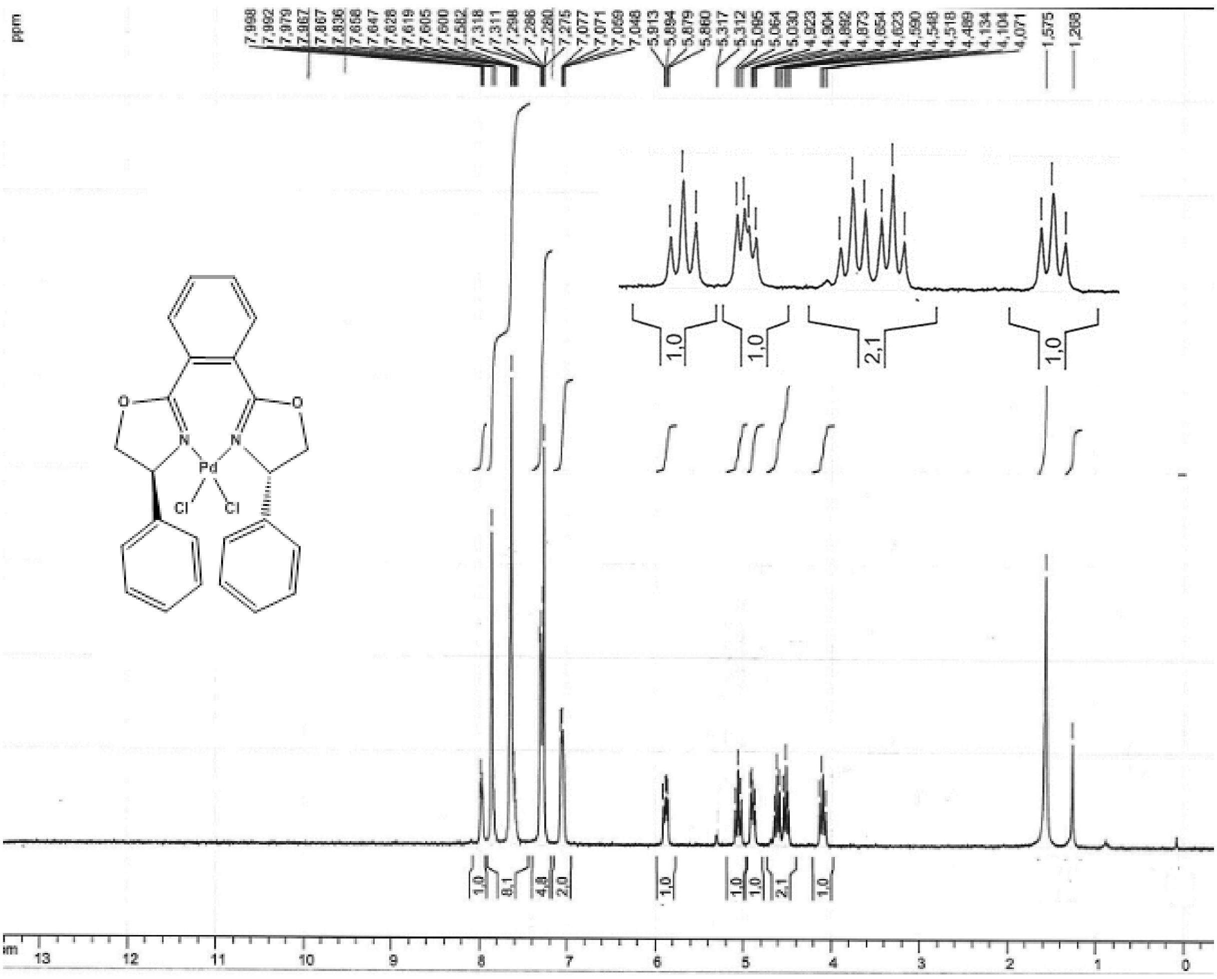
ppm
7,998
7,992
7,979
7,967
7,867
7,836
7,658
7,647
7,628
7,619
7,605
7,600
7,582
7,318
7,311
7,298
7,286
7,280
7,275
7,077
7,071
7,059
7,048
5,913
5,894
5,879
5,860
5,317
5,312
5,095
5,064
5,030
4,923
4,904
4,892
4,873
4,854
4,623
4,590
4,548
4,518
4,489
4,134
4,104
4,071
1,575
1,268
O
N
Pd
Cl
1,0
1,0
2,1
1,0
1,0
8,1
4,8
2,0
1,0
1,0
1,0
2,1
1,0
13
12
11
10
9
8
7
6
5
4
3
2
1
0

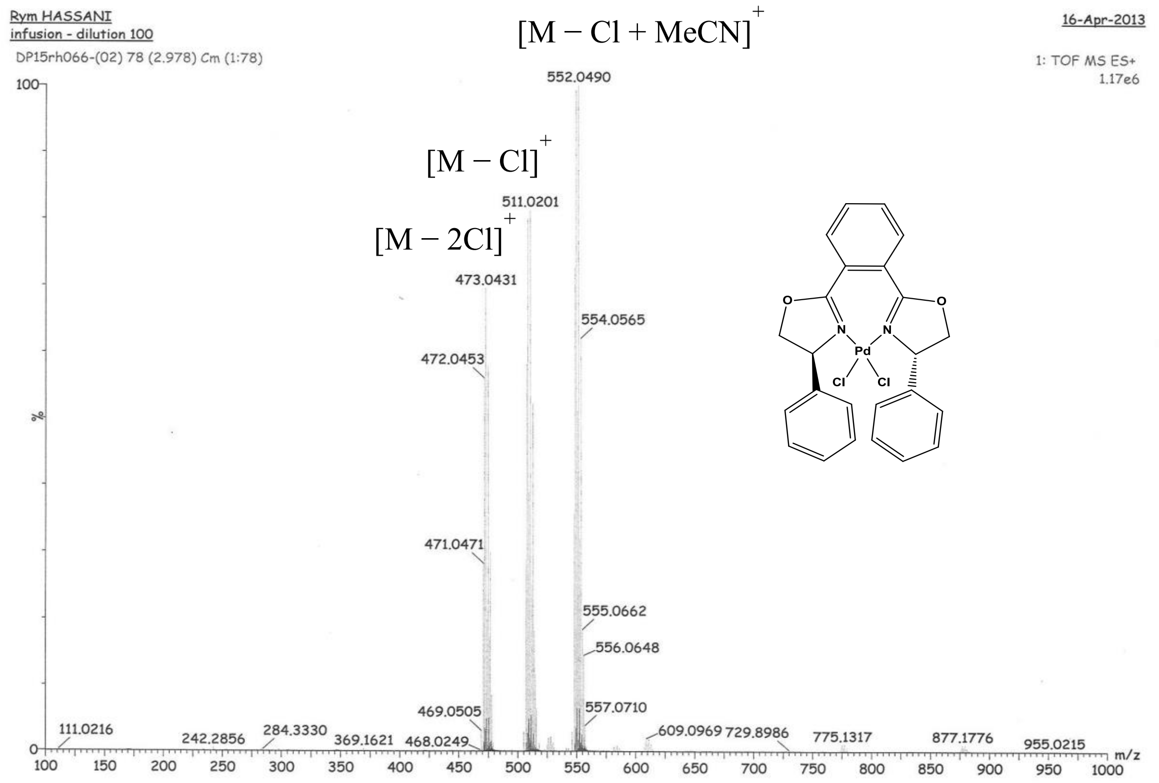
Rym HASSANI
infusion - dilution 100
DP15rh066-(02) 78 (2.978) Cm (1:78)
16-Apr-2013
1: TOF MS ES+
1.17e6
[M − Cl + MeCN]+
[M − Cl]+
[M − 2Cl]+
552.0490
511.0201
473.0431
554.0565
472.0453
471.0471
555.0662
556.0648
557.0710
469.0505
111.0216
242.2856
284.3330
369.1621
468.0249
609.0969
729.8986
775.1317
877.1776
955.0215
%
m/z
O
N
Pd
Cl
